# Vernalization Mediated Changes in the *Lolium perenne* Transcriptome

**DOI:** 10.1371/journal.pone.0107365

**Published:** 2014-09-16

**Authors:** Cristiana Paina, Stephen L. Byrne, Cristian Domnisoru, Torben Asp

**Affiliations:** 1 Department of Molecular Biology and Genetics, Science and Technology, Aarhus University, Slagelse, Denmark; 2 k4C Consulting, Eden Prairie, Minnesota, United States of America; NIH, United States of America

## Abstract

Vernalization is a key requirement for the induction of flowering in perennial ryegrass (*Lolium perenne* L.). The transcriptome of two genotypes with contrasting vernalization requirement was studied during primary (vernalization and short day conditions) and secondary induction (higher temperature and long day conditions) using an RNA-Seq approach. This revealed transcripts with expression profiles indicative of a role in floral induction, both in the promotion and repression of flowering. We observed similarities and specific differences between the two genotypes related to cold response, carbohydrate metabolism, and photoperiod regulation. Components of the photoperiod pathway showed regulation during vernalization, pointing to possible interactions between elements of the photoperiod and vernalization pathways. The results provide a global picture of the processes ongoing during the transition from vegetative to reproductive phase of perennial ryegrass genotypes with and without a vernalization requirement.

## Introduction

Plants from temperate regions have developed complex mechanisms for the change from vegetative to reproductive growth in order to time this transition to occur when conditions for pollination and seed development are optimal. The control over the timing of flowering is achieved through intricate regulatory networks in tight connection with environmental signals. As many other plants from temperate regions, perennial ryegrass developed a dual requirement for the induction of flowering [1]. It requires a primary induction, represented by several weeks of low temperature (vernalization) accompanied by short days. This is followed by the secondary induction, which starts when the temperature and the day length increase.

Floral induction has been investigated on the molecular level in both dicots and monocots and genes playing a role in the promotion or repression of flowering have been elucidated (reviewed in [2]–[5]). The different flowering pathways interact and converge on a number of key regulators, forming a complex flowering induction regulatory network to ensure reproductive success. Environmental and endogenous signals are integrated by the floral induction pathways. The environment sensing pathways rely on external signals which contribute to the seasonal regulation of flowering. The photoperiod pathway relates floral induction to changes in the day length, in a tight interaction with components of the circadian clock. The vernalization pathway integrates temperature related signals to time the transition to flowering after winter. Although the mechanism shares some similarities, the components of the vernalization pathway differ between monocots and dicots [6]. The endogenous floral induction pathways integrate plant derived signals to mediate the transition to reproductive phase. As part of this, the autonomous pathway integrates development related signals to promote flowering. The activity of miRNA156 and miRNA172 was shown to regulate flowering time, and was related to plant age [7], [8]. Phytohormones regulate many growth and development related processes in plants, being involved in the phase change as well, with gibberellins (GA) having a special role in the induction of flowering in many plant species [9]. A wide range of metabolic compounds regulate a diversity of processes related to plant growth and development, being also involved in the transition from vegetative to reproductive phase of plants. Specific modifications and interactions were described at the genetic, epigenetic, as well as at the protein level, within and between the floral induction pathways. However, there are several missing nodes and the complexity of regulatory networks is still to be unraveled.

The vernalization requirement has previously been studied in perennial ryegrass using genotypes with contrasting requirements for the induction of flowering [10], [11]. Falster is a perennial ryegrass ecotype from Denmark that has a strong vernalization requirement. It requires at least eight weeks of vernalization in order to flower. Veyo is a synthetic variety originating from central Italy that has no vernalization requirement. Veyo plants have the ability to flower before and during primary induction and show a strong response to long day conditions. The specific primary and secondary induction requirements of the two genotypes were previously described in detail [10]–[12]. These two genotypes are the grandparents of the Vrn-A mapping population developed to study the vernalization response. Based on this mapping population, five vernalization response quantitative trait loci (QTL) were identified [11], [13].

The development of next-generation sequencing technologies has opened up new possibilities to shed light on the intricate networks underlying different processes in living organisms. In particular, it enables global transcriptome studies to now be performed in non-model species that have lacked many of the array based assays so successfully used to study gene expression in model species. Here we took advantage of the high throughput sequencing technologies to study vernalization mediated changes in the transcriptome of perennial ryegrass and relate these changes to what we know from model species. The specific aims of this study were: i) to identify vernalization mediated candidate genes in perennial ryegrass by clustering transcripts regulated through the floral induction process, and ii) to identify molecular pathways differentiated between genotypes with and without a vernalization requirement.

## Materials and Methods

### Plant material

Both Falster and Veyo genotypes were subjected to clonal propagation. Ten newly formed tillers were selected for each plant and planted in 1 liter pots, paying special attention to assure the same developmental stage for all tillers and both genotypes. The plants were grown in the greenhouse for 75 days and were afterwards transferred to a climate chamber for 58 days under simulated autumn conditions, with 15°C and 8 hours day length. The vernalization treatment was carried out for nine weeks, at a temperature of 6°C and 8 hours day length. Leaf material was collected before the start of vernalization, after two days, four and nine weeks of vernalization. Enriched meristem was harvested at nine weeks of vernalization. During primary induction, the plant material was harvested between 9–10 am. The secondary induction was carried out at a temperature of 20°C and 16 hours day length. Leaf and enriched meristem samples were collected after one and seven days of long day conditions, between 1.30–5 pm. The light irradiance was measured throughout the experiment as having a mean of 51.72 µEinstein/m^2^/min, measured at soil level. In case of each genotype, special attention was paid during sampling in order to ensure uniformity between the samples. The upper two thirds of the most recently developed and fully expanded leaf was harvested at each time point. For enriched meristem samples, 2 mm from the basal node was harvested to include the apical meristem, removing as much of the surrounding leaf tissues as possible. All plants were in vegetative stage throughout the course of the experiment, showing no visible signs of heading. The heading date of the plants was not recorded within this experiment. However, a previous study reported the flowering time for both Falster and Veyo genotypes under similar conditions [11] and the heading date of the plants in the present experiment was expected to be similar to these reports. Table 1 summarizes the sample collection time points and the harvested plant material.

**Table 1:** Plant material harvested from the Falster and Veyo genotypes and sample collection time points.

Collection time points	Plant material harvested
before the start of vernalization	leaf
2 days of vernalization	leaf
4 weeks of vernalization	leaf
9 weeks of vernalization	leaf, enriched meristem
1 long day	enriched meristem
7 long days	leaf, enriched meristem

Total RNA was extracted using the RNeasy Plant Mini kit (Qiagen) following the manufacturer's protocol. The RNA quality and concentration were assessed with the Agilent RNA 6000 Nano kit on the Agilent 2100 Bioanalyzer (Agilent Technologies). From each time point, one sample representing equal amount of plant material pooled prior to RNA extraction from four individual plants, was prepared for sequencing.

### mRNA-Seq library preparation for RNA-Seq and Illumina Genome Analyzer II sequencing

10 µg of total RNA from each sample was used for duplexed mRNA-Seq library construction, combining the protocol supplied with the mRNA-Seq Sample Prep Kit (Illumina, protocol version 1004898 Rev. D, September 2009) and the Multiplexing Sample Preparation Oliogonucleotide Kit. mRNA was purified using poly-T oligonucleotide-attached magnetic beads and fragmented using divalent cations under elevated temperature. The RNA fragments were then copied into first strand cDNA using reverse transcriptase and random hexamer primers, followed by second strand cDNA synthesis using DNA Polymerase I and RNaseH. The short cDNA fragments were “end-repaired” using T4 DNA polymerase and Klenow DNA polymerase and a single A base was added to the cDNA fragments by using 3′-to-5′ exonuclease. Illumina adaptors from the Multiplexing Sample Preparation Oliogonucleotide Kit were ligated and fragments of approximately 200 bp in size were gel purified. The libraries were finally duplexed (index #6: GCCAAT or index #12: CTTGTA) and enriched by PCR for 18 cycles using the primers from the Multiplexing Sample Preparation Oliogonucleotide Kit.

Equimolar amounts of each of the libraries were pooled, diluted in buffer EB (QIAGEN) to 10 nM, denaturated with 2 N NaOH to a final DNA concentration of 1.0 nM, further diluted to 10 pM with pre-chilled Hybridization buffer (Illumina), and subsequently loaded into the lanes of a flow cell (v4). Three technical replicates were loaded for each sample. A single lane of the Multiplexing PhiX Control library (Illumina) was included in the flow cell and used to estimate phasing and matrix correction for all other lanes. Clustering was conducted on the Illumina Cluster Station followed by 76+7+76 cycle sequencing on the Illumina Genome Analyzer (version IIx, SCS 2.6.26(RTA 1.6.47.1) using the Genomic DNA Sequencing Primer, the Index Seq Primer, and the Multiplexing Rd2 Seq Primer, in combination with clustering and sequencing kits (SBS v4), all supplied by Illumina. RTA output was analyzed using the CASAVA software (version 1.6, Illumina), generating pass filtering FastQ files with Qphred +64 quality values. Single end reads with a length of 50 bp were generated. The reads were deposited in the EMBL-EBI ArrayExpress Archive, under accession number E-MTAB-2623. The quality of the reads was assessed using FastQC [14].

### Data processing

Separate *de novo* assemblies were constructed for each genotype using Trinity assembler, release 28.07.2011 [15]. The assemblies were filtered for a minimum sequence length of 100 bp (EMBL-EBI ArrayExpress, accession no. E-MTAB-2623). We compared our assemblies to the publicly available *Brachypodium distachyon* coding sequence data set version 1.0 available 14.09.2011 [16], in order to evaluate the completeness of the assemblies. BLASTn version 2.2.25 [17] was used with an e-value cutoff of 10^e-06^ and only hits with at least 100 bp overlap were considered.

The reads were mapped to the assembled transcripts in order to assess the expression level at the different time points. RSEM version 1.1.11 [18] was used to generate the read count data for the assembled transcripts, separate for each time point, sample type, and each genotype. A maximum of one mismatch (*–bowtie-n 1*) was allowed in the seed region of the reads.

The option for no biological replicates from DESeq version 1.4.1 [19] was used for differential expression analysis, with ‘*method = blind*’. Pairwise comparisons were carried out between all the selected time points. DESeq highlighted a set of transcripts for each pairwise comparison. These transcripts will be further referred to as differentially expressed transcripts (EMBL-EBI ArrayExpress, accession no. E-MTAB-2623). The variance stabilized data obtained from DESeq was used as input for clustering, and for constructing multidimensional scaling plots using R [20]. The transcripts showing differential expression at any time point during floral induction were clustered using a K-means clustering algorithm. The samples in this experiment were taken at various time points through primary and secondary induction and the distance in time between the sampling points varies. To take this into consideration, we have augmented the *K*-means clustering method with an algorithm developed to take into account the distance in time between the collection time points (detailed in Supplementary Material S1). In order to compare the differentially expressed transcript sets identified in the two different genotypes, BLASTn (E-value threshold 10^e-10^) was used to map the transcripts back to a common reference transcriptome of an inbred *L. perenne* genotype, generated from a combination of root, stem, leaf sheath, leaf and meristem samples [21]. The differentially expressed transcripts were annotated using Blast2GO [22], [23]. An E-value threshold of 10^e-06^ was used for the BLASTx search, and 10^e-10^ for the annotation, with a cut-off value of 55 and a GO weight Hsp-hit value of 20. The enrichment analysis for the differential gene ontology term distribution was performed with a *p*-value significance cut-off value of 0.01.

### RT-PCR validation

The experimental setup described above under ‘Plant Material’ was repeated to obtain material for RT-PCR validation of the differentially expressed transcripts. Plant material from five biological replicates was collected at each time point, from both genotypes. Total RNA was extracted as described earlier and quantified using the Quant-iT RiboGreen RNA Reagent and Kit (Life Technologies, Invitrogen) according to the manufacturer's instructions. cDNA synthesis was carried out using random nanomer primers and SuperScript II (Invitrogen, Life Technologies) as described in Supplementary Material S2. A random set of 42 transcripts identified as differentially expressed in leaf samples (21 in Falster and 21 in Veyo) were chosen for validation using quantitative RT-PCR. A preliminary draft shotgun assembly of the perennial ryegrass genome [24] was used to design primers across two exons to avoid amplification resulting from genomic DNA contamination of the samples. Primers were designed with Primer3 software [25], to amplify a 150–200 bp product (details in Supplementary Material S2). The specificity of the primers and the amplification product size were verified on 1.5% agarose gel stained with ethidium bromide.

Quantitative RT-PCR reactions were set up using SYBRGreen PCR Master Mix (Life Technologies, Applied Biosciences) according to the manufacturers recommendations, and were run on the ViiA7 Real-Time PCR System Instrument (Life Technologies, Applied Biosciences) (see Supplementary Material S2 for full reaction and cycling parameters). The YT 521-B-like protein family protein was used as a housekeeping gene for reactions with annealing temperature of 64°C, and the eukaryotic elongation factor alpha was used as a housekeeping gene for reactions with annealing temperature of 59°–60°C [26], [27]. Each time point had five biological replicates and three of these were performed in duplicates in order to assess technical variability. Amplification efficiencies associated with each primer set were determined using a dilution series of template cDNA. The data analysis was performed with Q-Gene [28]. The results obtained when using housekeeping genes for normalization were identical to those obtained when using total RNA for normalization.

## Results

### Falster and Veyo transcriptome assemblies

A total of 111,016,044 reads of 50 bp were generated for the genotype Veyo and a total of 108,370,252 reads were generated for Falster. Separate transcriptome assemblies were constructed for each genotype using all their available reads. The Veyo assembly had a total size of 60 Mb, 50% of which was present in contigs of at least 711 bp (Table 2). The Falster assembly had a total size of 63 Mb, 50% of which was present in contigs of at least 655 bp (Table 2). The longest assembled contigs in Falster and Veyo assemblies have 15,334 bp and 15,204 bp, respectively. Both correspond to the *Brachypodium distachyon* auxin transporter BIG-like protein transcript, one of the largest transcripts in *Brachypodium* with a size of 13,221 bp. This demonstrates the capacity of the assembly algorithm to reconstruct large transcripts using short-read sequence data. To assess the completeness of the assemblies, we compared them to the *Brachypodium distachyon* coding sequence data set consisting of 32,255 entries. A total of 27,634 *Brachypodium* sequences (85.67%) had a significant hit in the Falster transcriptome assembly and 27,685 (85.83%) had a significant hit in the Veyo transcriptome assembly.

**Table 2:** Trasncriptome assembly results.

Genotype	Min. contig length (bp)	N50 (bp)	Mean contig length (bp)	Max. contig length (bp)	Sum (no. of total bp in the assembly)	Total no. of contigs
Falster	100	655	372	15,334	63,810,000	171,448
Veyo	100	711	383	15,204	60,080,000	156,832

### Differential expression

**Figure 1:**
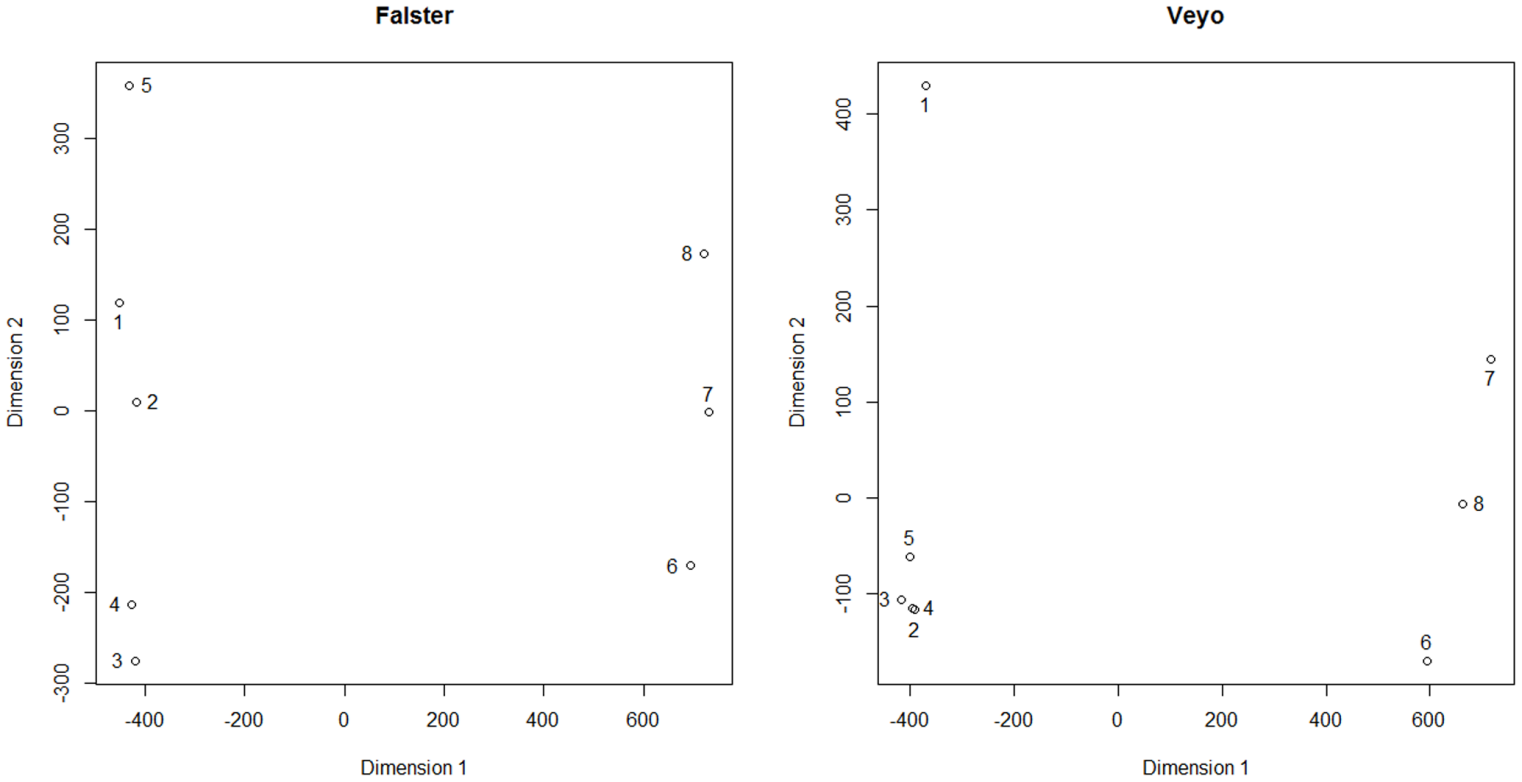
Multidimensional scaling plots of the Falster and Veyo transcriptome assemblies. The first dimension illustrates grouping based on sample type. The second dimension illustrates grouping based on the sample collection time points (stage of induction). The numbers illustrate the samples as follows: 1 =  leaf samples collected before the start of vernalization; 2 =  leaf samples collected after two days of vernalization; 3 =  leaf samples collected after four weeks of vernalization; 4 =  leaf samples collected after nine weeks of vernalization; 5 =  leaf samples collected after seven long days; 6 =  enriched meristem samples collected after nine weeks of vernalization; 7 =  enriched meristem samples collected after one long day; 8 =  enriched meristem samples collected after seven long days.

Multidimensional Scaling (MDS) plots were generated for each genotype based on the variance stabilized expression data from DESeq in order to determine sample relationships (Figure 1). A clear separation was observed between leaf and enriched meristem samples on the first dimension, for both genotypes. This was expected considering the contrasting biological processes ongoing in leaf and meristem. The second dimension groups samples based on stage of induction. On this dimension we observed clear differences between Falster and Veyo plants that fit with their contrasting vernalization requirement. As Falster has a minimum vernalization requirement of eight weeks, we expected the transcriptome profile to change during the course of vernalization and show some distinction from the profile after only two days of vernalization. This is what was observed. The samples from four and nine weeks of vernalization showed clear separation from the sample collected after only two days of vernalization, suggesting significant changes in the transcriptome during cold treatment. This is in clear contrast to the genotype Veyo, which has no vernalization requirement. The fact that all Veyo leaf samples collected during vernalization clustered tightly together may indicate that the greatest changes in the transcriptome of this genotype during this treatment are related to the initial cold stress, and it doesn't undergo large scale alterations as the cold period progresses.

**Figure 2:**
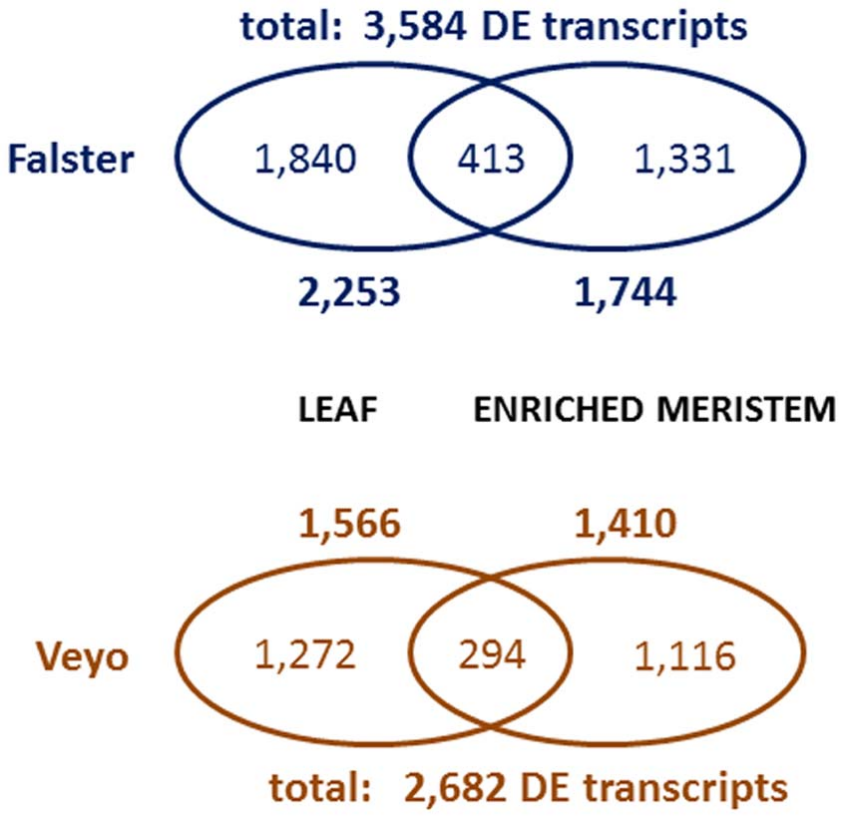
The number of differentially expressed transcripts common and specific for each sample type.

In order to assess the expression level of each assembled transcript at the different time points, the reads from each sample were mapped onto their respective genotype specific assemblies. In the case of each sample, more than 90% of the reads mapped onto the assembled transcripts. In a series of pairwise comparisons between all the selected time points, we identified 2,253 differentially expressed transcripts in Falster leaf and 1,744 in enriched meristem samples. We identified 1,566 differentially expressed transcripts in Veyo leaf and 1,410 in enriched meristem samples. There were 413 Falster and 294 Veyo transcripts differentially expressed in both leaf and enriched meristem (Figure 2). In some cases, different putative splice variants were present in leaf compared to enriched meristem samples. Furthermore, several transcripts were identified as differentially expressed between more than two time points, indicating significant modulation throughout floral induction.

In general, there was a higher number of differentially expressed transcripts in Falster compared to Veyo (Figure S1). For Falster leaf samples, the highest number was observed in the pair wise comparison between four weeks of vernalization and seven long days. Veyo leaf samples had the highest number of differentially expressed transcripts between the time point before vernalization and seven long days, the only case with a higher number compared to Falster. In enriched meristem, Falster had the highest number of transcripts differentially expressed between nine weeks vernalization and seven long days, while Veyo between nine weeks vernalization and one long day, the only case higher than Falster. It was expected that there would be a high number of transcripts differentially expressed when going from autumn growing conditions to vernalization inducing conditions due to the significant change in temperature, and this is what we see in both Falster and Veyo genotypes. Several differentially expressed transcripts may be involved in a rapid response to cold stress. However, an extended period of cold (eight weeks) is required in order to fulfill the vernalization requirement in Falster. This would mean that genes involved in the promotion or repression of flowering through the vernalization pathway would undergo changes in expression throughout vernalization. We observed a higher number of transcripts differentially expressed during vernalization in Falster in comparison to the non-vernalization requiring genotype, Veyo.

Comparing the differentially expressed transcripts from the two different genotypes, we would expect a set of transcripts to be present in both. The common set can be related to stress genes, cold sensing and acclimation genes, photoperiod response genes. We found a total of 932 Falster differentially expressed transcripts (470 from enriched meristem and 587 from leaves) showing changes in expression level in Veyo plants also (Supplementary Material S3).

### RT-PCR validation of differentially expressed transcripts

38 out of the 42 randomly selected transcripts showed specific amplification with the tested primers. A total of 19 of these sequences were identified as differentially expressed in Falster and 19 in Veyo leaf samples. The differentially expressed transcripts were identified through pairwise comparisons between all our collection time points. Some of these sequences were identified as differentially expressed in more than one pairwise comparison (between more than two time points), showing thus more than one change in expression level throughout the experiment. Out of the 38 tested sequences, 33 (86.85%) had at least one change in expression level confirmed as significant (Supplementary Material S4).

### Annotation of differentially expressed transcripts

Between 46–56% of the differentially expressed transcripts were annotated using Blast2GO. Putative descriptions and functions were assigned to the transcripts predominantly based on annotations from *Hordeum vulgare* and *Brachypodium distachyon*. The complete list of differentially expressed transcripts along with the BLASTx results and annotation is presented in Supplementary Material S3.

Fisher's Exact test from Blast2GO was used to determine if any gene ontology (GO) terms were over- or under-represented in the various sets of differentially expressed transcripts. A total of 33 GO terms were enriched when comparing the differentially expressed transcript sets from leaf samples of the two genotypes (Figure S2). Out of these, 14 were overrepresented in Falster, including terms related to dephosphorylation, transmembrane transport, and organelle envelope. Transcripts assigned to peptidyl-amino acid modification were present only in Falster. In Veyo leaf samples, 23 GO terms were enriched and were related to cellular aromatic compound, metabolic process, regulation of nucleotide metabolic process, regulation of catabolic process, and RNA polymerase activity. A number of 11 GO terms grouped sequences specific to Veyo leaf samples, including regulation of Rab protein signal transduction and Rab GTPase activity, positive regulation of hydrolase, and catalytic activity. A total of 28 GO terms were overrepresented when comparing the enriched meristem samples (Figure S3). Out of these, 27 GO terms were overrepresented in Falster and were related to ATP binding, nucleotide binding, mRNA metabolic process, and to membranes and mitochondria. A number of 10 GO terms grouped only Falster transcripts, related to mitochondrial processes, RNA-methylation, and tau-protein kinase activity. Only one GO term, acetylglucosaminyltransferase activity, was overrepresented in Veyo enriched meristem samples compared to Falster.

In the enrichment analysis of Falster transcripts differentially expressed between the non-inductive time point and the time points during vernalization, compared to the complete set of differentially expressed transcripts from Falster leaf samples, we observed an overrepresentation of GO terms related to reproduction and development (Figure S4). This was not the case in Veyo.

### Expression profiling

**Figure 3:**
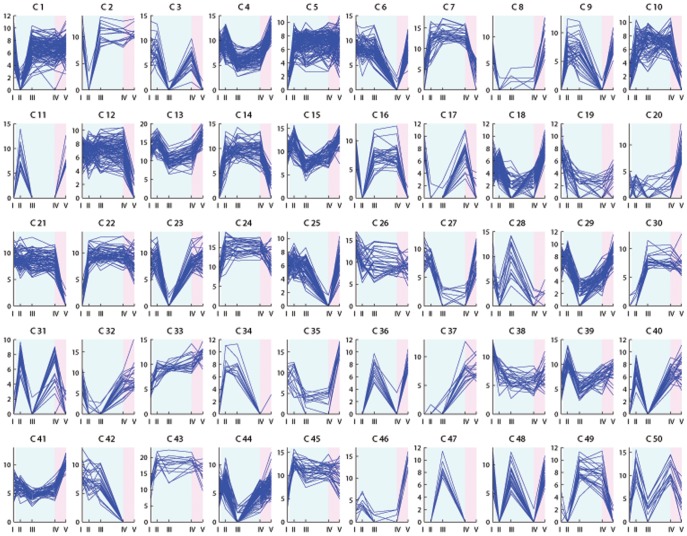
K-means clustering analysis of transcripts differentially expressed in Falster leaf samples. K = 50 clusters. The *x* axis presents the sample collection time points: I =  autumn conditions, before the start of vernalization; II = 2 days of vernalization; III = 4 weeks of vernalization; IV = 9 weeks of vernalization; V = 7 days of long day conditions. The *y* axis corresponds to the variance stabilized data from DESeq. The blue background illustrates vernalization treatment, the pink background illustrates long day conditions.

**Figure 4:**
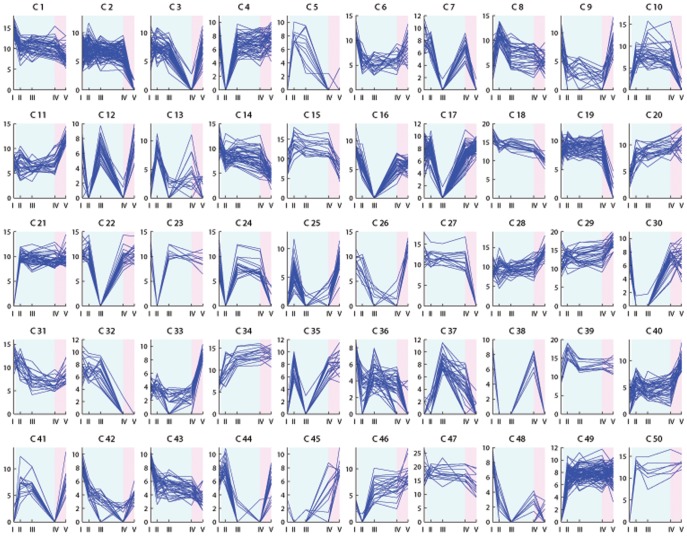
K-means clustering analysis of transcripts differentially expressed in Veyo leaf samples. K = 50 clusters. The *x* axis presents the sample collection time points: I =  autumn conditions, before the start of vernalization; II = 2 days of vernalization; III = 4 weeks of vernalization; IV = 9 weeks of vernalization; V = 7 days of long day conditions. The *y* axis corresponds to the variance stabilized data from DESeq. The blue background illustrates vernalization treatment, the pink background illustrates long day conditions.

**Figure 5:**
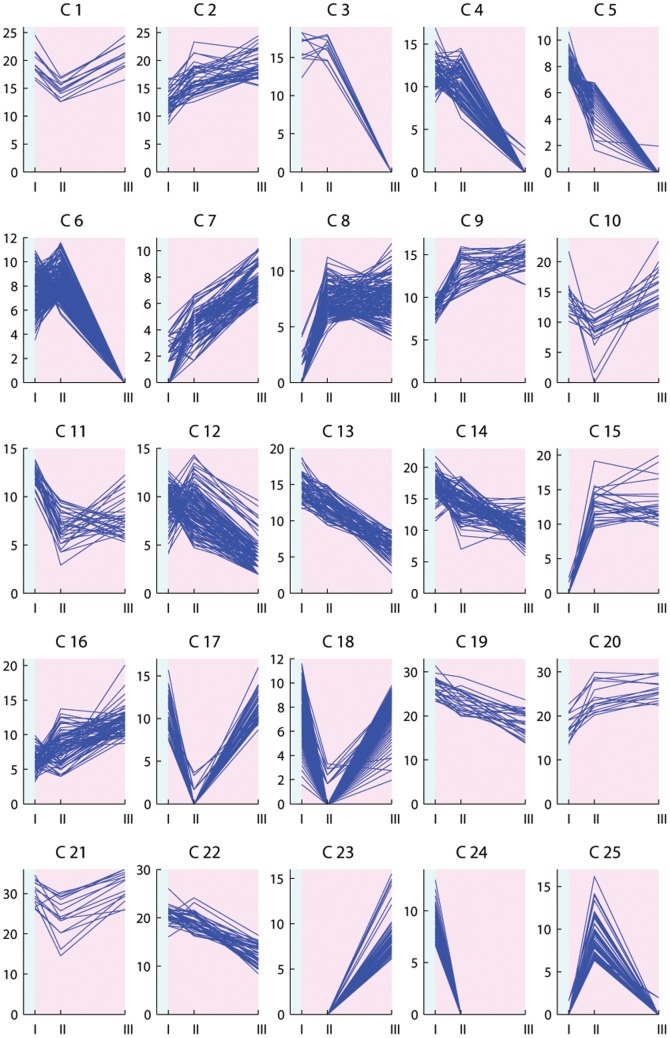
K-means clustering analysis of transcripts differentially expressed in Falster enriched meristem samples. K = 25 clusters. The *x* axis presents the sample collection time points: I = 9 weeks of vernalization; II = 1 long day; III = 7 days of long day conditions. The *y* axis corresponds to the variance stabilized data from DESeq. The blue background illustrates vernalization treatment, the pink background illustrates long day conditions.

**Figure 6:**
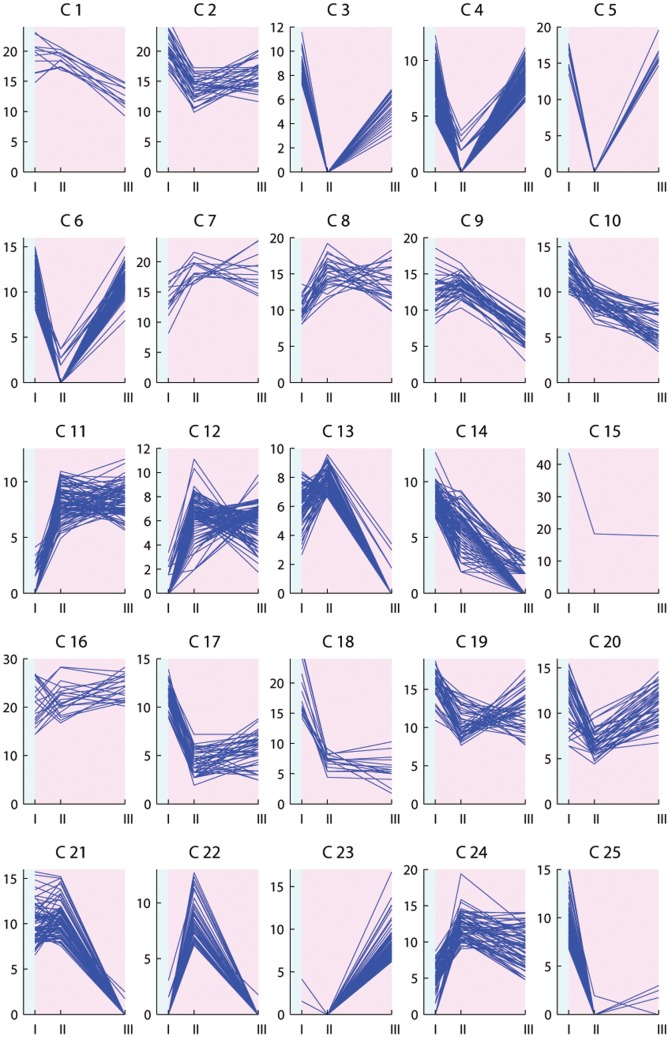
K-means clustering analysis of transcripts differentially expressed in Veyo enriched meristem samples. K = 25 clusters. The *x* axis presents the sample collection time points: I = 9 weeks of vernalization; II = 1 long day; III = 7 days of long day conditions. The *y* axis corresponds to the variance stabilized data from DESeq. The blue background illustrates vernalization treatment, the pink background illustrates long day conditions.

The expression profiles of the differentially expressed transcripts were investigated through floral induction and are illustrated in Figures 3–6. Clustering the differentially expressed transcripts based on expression profiles allowed to distinguish and focus on groups of genes indicative of a potential role in vernalization mediated floral induction.

The shift from autumn conditions to vernalization (sudden drop in temperature from 15°C to 6°C), and the shift from vernalization to secondary induction (sudden increase in temperature from 6°C to 20°C, and in photoperiod from 8 hours to 16 hours) can result in plant stress and lead to the rapid up- or down-regulation of transcripts. Strong up- or down-regulation at only two days of vernalization in the case of leaf samples, or only at one long day in case of enriched meristem, is indicative of transcripts with a role in stress response (Fig. 3 cluster 2; Fig. 4 clusters 4, 23; Fig. 5 clusters 1, 17, 18, 25; Fig. 6 clusters 4, 5, 6, 13, 22). Transcripts up-regulated at two days of vernalization and maintaining this expression until the end of vernalization, but being down-regulated after the transition to long days, suggest a role in cold stress response and cold acclimation (Fig. 3 clusters 7, 10, 14, 24, 43; Fig. 4 clusters 10, 15, 47).

While cold response transcripts are expected to show a strong regulation from the beginning of cold treatment, several weeks are necessary to fulfill the vernalization requirement in Falster. We would expect genes involved in mediating vernalization to show modulation through the entire vernalization period, and not after only two days. We identified clusters with relevant profiles in both Falster and Veyo plants. A number of transcripts were differentially expressed in both genotypes, showing similar or different expression patterns, but did not have a BLAST hit in public databases (Supplementary Material S3). Four such transcripts without a hit in public databases were included in the randomly selected set for RT-PCR testing and were validated.

Vernalization is expected to down-regulate genes involved in the repression of flowering. Some flowering repressors may show a gradual decrease in expression level through the cold treatment (Fig. 3 cluster 26; Fig. 4 clusters 1, 18, 42, 43). Others would need a longer period of cold (vernalization) in order to be down-regulated (Fig. 3 clusters 19, 42; Fig. 4 clusters 32, 48). Vernalization is also expected to down-regulate genes involved in the activation of floral repressors (Fig. 3 clusters 32, 27, 29, 35, 6, 25; Fig. 4 clusters 26, 3). On the other hand, some of the genes involved in the promotion of flowering in response to vernalization would gradually increase in expression level through the nine weeks of vernalization, and possibly maintain this level through the initial stages of secondary induction (Fig. 3 cluster 33; Fig. 4 clusters 20 and 34). Other flowering promoters may require a longer period of cold and would therefore have a later response during vernalization (Fig. 3 clusters 30, 37; Fig. 4 cluster 45). Falster cluster 30 (Fig. 3) groups 25 transcripts modulated by vernalization in a manner specific to this genotype. Thirteen of these transcripts had hits in public databases and were annotated (Supplementary Material S3). Their expression profile is highly suggestive of potential involvement in vernalization mediated floral induction, showing a strong increase in expression level at four weeks of vernalization that is maintained into secondary induction. Transcripts involved in the activation of the flowering promoters through the vernalization pathway are also expected to be up-regulated during vernalization (Fig. 3 clusters 34, 49, 5, 22; Fig. 4 clusters 5, 37, 21, 49, 50).

Transcripts up-regulated only at seven long days (Fig. 3 clusters 20, 46; Fig. 4 clusters 11, 45; Fig. 5 cluster 23; Fig. 6 cluster 23) would probably be involved in processes specific to secondary induction. However, some could correspond to genes involved in the transition from vegetative to reproductive phase activated through flowering promoters induced by vernalization, or activated through the photoperiod pathway induced by long days.

## Discussion

This study set out to uncover the transcriptome profiles of perennial ryegrass through floral induction in genotypes with a contrasting requirement for vernalization. The genotype Falster, from Denmark, has a strong vernalization requirement, while the genotype Veyo, from Italy, has no vernalization requirement. This represents excellent material for studying the transcriptome through vernalization and the transition to long days. It was envisaged that we would be able to identify transcripts potentially involved in mediating the vernalization requirement of the Falster genotype by taking advantage of the resolution provided by RNA-Seq. The sample collection time points were chosen to allow differentiation between stress responses and processes specific to vernalization and the transition to long days. The plants were exposed to a period of 58 days of short day conditions before the start of vernalization treatment in order to differentiate between transcripts responding to short photoperiod and transcripts responding to low temperature, i.e. vernalization. In some temperate grasses, short day conditions were reported to substitute for vernalization [1], [29], [30]. Although previous studies tested the requirements of the two genotypes and their flowering response to different primary and secondary induction treatments [10], [11], no record is available referring to their response solely to short day conditions with respect to flowering.

### Transcripts rapidly regulated in response to cold

As vernalization involves a period of low temperature, many of the differentially expressed transcripts will be simply related to the response to cold and may not be directly involved in the vernalization flowering pathway. It was envisaged that many cold responsive genes would be differentially regulated at two days of vernalization. Two days is not long enough to fulfill the vernalization requirement in Falster [10] and therefore, these transcripts alone are most likely not capable of promoting flowering in response to vernalization. At two days of vernalization, we did find an enrichment of stress response genes in both genotypes. Some cold responsive transcripts were similarly regulated in both genotypes. Others showed a genotype specific regulation, suggestive of differences in the cold acclimation processes between perennial ryegrass genotypes that differ in both latitudinal origin and in their requirement for vernalization. Transcripts corresponding to the ice recrystallization inhibition (IRI) proteins, cold-induced and cold response (COR) proteins, dehydrins (DHN), and late embryogenesis abundant (LEA) proteins, as well as aquaporin, thiamine, and proline transcripts were all found to be up-regulated at two days of vernalization (Fig. 3 clusters 7, 14, 24; Fig. 4 clusters 15, 47), in agreement with previous studies [31]–[39]. These are all typical transcripts that we would expect to be up-regulated after such a dramatic and rapid shift in growth temperature, from 15° to 6°C. Conversely, we also see transcripts down regulated in response to the cold (Fig. 3 clusters 8, 27; Fig. 4 clusters 9, 26), many of which are involved in key metabolic processes. This was expected since growth rates will slow down dramatically to preserve resources until more favorable conditions arrive. Rubisco activase and cysteine proteinase transcripts were down-regulated in both genotypes (Fig. 3 cluster 8; Fig. 4 cluster 9), in agreement with previous reports in barley [33].

Of interest in this class of transcripts that respond rapidly to cold are those that differ in their response between the northern and southern European genotypes. One such transcript was a delta-1-pyrroline-5-carboxylate synthetase that was up-regulated during cold treatment only in Falster, in both leaves and enriched meristem samples (Fig. 3 cluster 43; Fig. 5 cluster 19). The same expression profile was observed in vernalization-responsive barley seedlings [33]. Two phosphoethanolamine N-methyltransferase transcripts were up-regulated as well throughout vernalization only in Falster leaves (Fig. 3 cluster 43). The same was observed during cold acclimation of winter wheat, where it was suggested that, due to its role in choline and phospholipid metabolism, it plays an important role in maintaining the integrity of plasma membranes under low temperatures [40].

It could be envisaged that plants that have evolved a mechanism to ensure that flowering occurs after winter has passed, have also developed mechanisms to better cope with periods of cold, and there could be a link between cold acclimation and vernalization. In a study involving VERNALIZATION INSENSITIVE 3 (VIN3), the most up-stream gene in the *Arabidopsis* vernalization mediated flowering pathway, Bond et al. suggested no connections between the vernalization and cold acclimation pathways [41]. However, other studies in diploid and hexaploid wheat showed a correlation between VERNALIZATION 1 (VRN1), the key vernalization gene in grasses, and cold acclimation controlled by CBF, COR and LEA levels, describing different VRN1 alleles causing differences in freezing tolerance [42]–[44]. Up-regulated by vernalization, VRN1 acts in grasses as a promoter of phase change and flowering by down-regulating and maintaining the down-regulation of the flowering repressor VERNALIZATION 2 (VRN2) [45]. VRN2 blocks flowering before vernalization through repressing the expression of VERNALIZATION 3 (VRN3) [46]. VRN3 is the cereal homologue of the *Arabidopsis* FLOWERING LOCUS T (FT), an important floral pathway integrator responsible for promoting flowering [47], [48]. Previous studies reported the perennial ryegrass Falster and Veyo VRN1 expression profiles up to six weeks of vernalization, where the levels were reported to be higher in Veyo compared to Falster [12], [34], [49]. Our study identified a strong up-regulation in Falster leaves of the VRN1 transcript at nine weeks of vernalization and at seven long days compared to the first, non-inductive time point and two days of vernalization. The Falster VRN1 transcript showed a gradual up-regulation through vernalization, with a peak at nine weeks (Fig. 3 cluster 33, Supplementary Material S5). A comparative sequence analysis study involving the VRN1 alleles from Falster and Veyo revealed a 8.6 kb insertion in the first intron of the Falster allele, having no homology to the wheat or barley VRN1 gene sequences [49]. The Veyo VRN1 allele proved to be dominant, and it was hypothesized that this insertion/deletion might explain the differences in the vernalization response of the two genotypes. The Veyo VRN1 transcript was not significantly regulated during this study, which could be correlated with the lack of vernalization requirement of this genotype. Dillon et al. suggested the involvement of VRN1 in initiating the down-regulation of genes from the cold acclimation pathway [44]. Considering this scenario, the differences between the Falster and Veyo VRN1 alleles [49] could account, at least in part, for the differences observed in the cold response of these two genotypes. Another recent study showed that the presence of VRN1 is essential for the up-regulation of floral meristem identity genes through the gibberellic acid flowering pathway in wheat apices [50], pointing towards strong connections between VRN1 and other floral induction pathways.

We did not identify a significant regulation of VRN2 through this experiment. However, since VRN2 was shown to be down-regulated under short day conditions [51], [52], this may be due to a potential significant drop in VRN2 expression levels already during the 58 days of short days during fall conditions which preceded the vernalization treatment. Also, no transcript corresponding to VRN3 was identified as differentially expressed in either genotype through vernalization and up to seven long days.

### Components of the circadian clock and photoperiod pathway regulated during vernalization

Components of the photoperiod pathway regulate flowering time in a tight interaction with components of the circadian clock (reviewed in [4]). Plants with no vernalization requirement adjust their flowering time according to the photoperiod. On the other hand, plants having a vernalization requirement adjust their flowering time according to changes in both temperature and photoperiod. We observed vernalization mediated regulation of some of the genes involved in the circadian clock and photoperiod flowering pathway.

One Falster and three Veyo transcripts with homology to GIGANTEA (GI) were identified as differentially expressed in leaf samples. GI is a key regulator of flowering time that was initially described in relation to its role in the photoperiod pathway and as a component of the circadian clock [53]. The Veyo sequences correspond to potentially different alleles and/or splice variants. All GI transcripts were gradually up-regulated through vernalization and maintained a high level at seven long days (Fig. 3 cluster 43; Fig. 4 cluster 34; Supplementary Material S5). Up-regulation of GI during cold treatment was described in previous studies, suggesting strong connections between the photoperiod and vernalization mediated flowering pathways, as well as the involvement of this gene in processes related to vernalization [54], [55]. Furthermore, Jung et al. showed GI to be involved in the regulation of miRNA172 in *Arabidopsis*, leading to up-regulation of FT and thus to flowering [56]. But GI can also directly activate FT in *Arabidopsis*, and was shown to interact with FT repressors, including SHORT VEGETATIVE PHASE (SVP) [57] which acts in the temperature pathway [58] and was recently shown to underlie a photoperiod flowering QTL [59]. The GI protein was shown to interact with SPINDLY in *Arabidopsis* and thus act within the gibberellic acid mediated flowering pathway [60]. Furthermore, GI was proposed to be involved in the sucrose signaling network [61] and to regulate the interconversion between sucrose and starch [62]. GI also interacts with and potentially regulates members of the salt stress tolerance pathway [63], [64]. GI appears thus as a point of convergence for several pathways. The observation that GI gradually increased in both genotypes as vernalization progressed may be suggestive of its involvement in primary induction. This increase in expression was maintained upon the switch to long days. A FLAVIN-BINDING KELCH REPEAT F-BOX (FKF1) transcript, also known as ADAGIO 3, was regulated in Veyo leaves in a similar manner to GI (Fig. 4 cluster 34). The *Arabidopsis* GI and FKF1 proteins were shown to have a light dependent interaction as components of the circadian clock as well as photoperiod flowering pathway promoters [65], [66]. They are part of a complex that mediates the degradation under long day conditions of CYCLING DOF FACTOR 1 (CDF1), a repressor of CONSTANS (CO) [67]. CO is a nuclear protein which promotes flowering under long day conditions through positive regulation of the floral pathway integrator FT. The GI-FKF1 complex regulates CO expression at the chromatin level, where FKF1 function was described to be dependent on GI [65]. In contrast to Veyo, FKF1 was not differentially expressed in the Falster genotype during vernalization, suggesting that regulation of FKF1 by vernalization is restricted to plants not having a strict vernalization requirement. The late flowering phenotype of the *fkf1 Arabidopsis* mutants was reported to be rescued by both vernalization and gibberellin treatment [66], pinpointing yet another connection between the floral induction pathways, and also, tempting to speculate that components of the vernalization pathway in Falster might compensate for this difference.

PSEUDO RESPONSE REGULATORS (PRR) regulate flowering time through the photoperiod pathway, in an interaction with components of the circadian clock [68]–[70]. One Falster transcript and two Veyo putative splice variants were annotated as PRR37. The Falster transcript and one of the Veyo transcripts had the same open reading frame (ORF) and were both up-regulated towards the end of vernalization and into long days (Fig. 3 cluster 33; Fig. 4 cluster 28; Supplementary Material S5). The other Veyo PRR37 transcript showed an 81 bp insertion which introduced a stop codon resulting in two ORFs within the transcript. This Veyo transcript had a different expression profile, being down-regulated only at two days of vernalization, which might indicate a cold stress response (Fig. 4 cluster 4). The expression of the cereal PRR37 gene, also known as PHOTOPERIOD 1 (PPD1), is increased by long days and leads to the photoperiod mediated activation of FT homologs, and to flowering (reviewed in [4]). PRR37 is most similar to *Arabidopsis* PRR7. *Arabidopsis* PRR5, PRR7, and PRR9 were shown to integrate low temperature signaling with the circadian clock, and it was proposed that they would be involved in both photoperiod and temperature regulation of floral induction [71], [72]. However, mutations in the barley PRR37 were reported to affect only photoperiodic flowering and not the circadian clock, indicative of functional differences between the *Arabidopsis* PRR7 and the cereal PRR37 genes [73]. We have found PRR37 transcripts up-regulated in leaves through primary induction with a further increase upon the shift to secondary induction. This was observed in genotypes both with and without a vernalization requirement. This is suggestive of potential involvement of PRR37/PPD1 in interactions with elements from the temperature dependent pathway.

The cereal homologue of the *Arabidopsis* LATE ELONGATED HYPOCOTYLE (LHY) and CCA1 is represented in our transcriptome assemblies by five Veyo and one Falster transcript. The Veyo LHY transcripts were down-regulated through vernalization and further into long days in leaves (Fig. 4 clusters 1, 18, 47; Supplementary Material S5). This correlates with the gradual up-regulation of PRR37, which was shown to down-regulate LHY [74]. LHY/CCA1 are negative regulators of the photoperiod flowering pathway under both short and long days [75] and regulate the flowering repressor SVP [76]. In *Arabidopsis*, LHY was shown to be involved in cold tolerance through regulating CBF and COR genes from the cold-response pathway [77]. We did not observe a significant regulation of LHY in Falster leaves through our experiment. However, LHY was down-regulated at one long day in both genotypes in enriched meristem (Fig. 5 cluster 1; Fig. 6 clusters 17, 20), which could represent a stress response to the sudden increase in temperature and photoperiod.

### Alterations in carbohydrate metabolism through vernalization

An important role for sucrose in controlling the transition to reproductive growth has been previously suggested [78]. Sucrose was shown to promote flowering, and proposed to function as a mobile signaling molecule in the flowering pathways, along with FT and gibberellins (reviewed in [79]). It was shown to up-regulate FT expression in *Arabidposis* but also to affect flowering independent of FT [80], [81]. Roldan et al. demonstrated the involvement of sucrose in the flowering of dark grown *Arabidopsis* plants, and also the ability of applied sucrose to alleviate or rescue the late flowering phenotypes of GI and CO, but also those of FCA, FPA, and FVE autonomous pathway mutants, with a stronger effect in dark grown plants [82]. But no effect of sucrose was observed on the FT mutants, supporting the FT regulation by sucrose. Quantitative differences in sucrose and starch levels associated with alterations in the carbohydrate metabolism were recently observed in the late flowering mutant of the INDETERMINATE 1 (ID1) autonomous pathway gene in maize [83]. Sucrose is also involved in cold response where, besides its role as a cryoprotectant, it was shown to act as a regulatory molecule and increase the expression of the cold responsive COR78 gene [84]. As a feedback loop, sucrose was shown to be regulated by cold response genes [85]. On the other hand, sucrose levels were shown to be under the control of the circadian clock [86]. Sucrose appears thus as another connection point between the cold acclimation and flowering pathways. We found components of the sucrose metabolic pathway that were differentially regulated during both primary and secondary induction. These were the genes for sucrose synthase (SS) SS1 and SS2. Sucrose synthase is a catalyst in the reversible reaction that converts sucrose and uridine-diphosphate into fructose and UDP-glucose [87]. Three putative splice variants of Falster SS2 showed up-regulation during vernalization in leaves (Fig. 3 clusters 14, 24, 45), while a SS1 transcript was down-regulated at two days of cold treatment and up-regulated in the second part of vernalization (Fig. 3 clusters 16). This is consistent with reports of increased SS expression in wheat during cold acclimation [88]. In contrast, a different SS-like transcript was up-regulated in Veyo leaves only after the shift to long days (Fig. 4 cluster 33), suggesting its connection to different processes. The differences observed in the leaves of the two genotypes in the regulation of SS through vernalization could be due to differences in the cold acclimation strategies of genotypes originating from northern and southern Europe. We cannot exclude however, that these differences could be related to their contrasting vernalization requirement and consequently to components of the flowering pathways. Transcripts corresponding to the SS1 gene were up-regulated in enriched meristems only after the shift to long days, and this occurred in both Falster and Veyo (Fig. 5 cluster 20; Fig. 6 cluster 16).

Rafinnose Family Oligosaccharides (RFOs) and fructans are important classes of water soluble carbohydrates that have a role as a reserve carbohydrate, in membrane stabilization, and as signaling molecules during stress response [89]. We observed changes in the expression of several transcripts involved in the metabolism of both RFOs and fructans. Galactinol synthase is involved in the biosynthesis of raffinose and has been proposed to play a role in cold tolerance in *Arabidopsis* and wheat [87], [90]. A transcript encoding galactinol synthase was up-regulated in Veyo leaves during vernalization (Fig. 4 cluster 15; Supplementary Material S5). Two putative splice variants of galactinol synthase were up-regulated in Falster after two days of cold treatment, and subsequently down-regulated during the remainder of vernalization (Fig. 3 cluster 15; Supplementary Material S5). So while the genotype from Mediterranean regions maintained its increased expression of galactinol synthase, the Nordic genotype Falster returned expression to pre-induction levels as vernalization progressed. Another enzyme, this time involved in raffinose degradation, was also regulated during the induction treatments. Two putative splice variants of alpha-galactosidase, the enzyme responsible for raffinose degradation, were down-regulated in Veyo leaves after two days vernalization and remained down-regulated throughout vernalization (Fig. 4 cluster 6). The down-regulation of this enzyme was shown to lead to raffinose accumulation and to enhance freezing tolerance in petunia, a half-hardy plant species [91]. Down regulation of alpha-galactosidase, together with increased galactinol synthase expression, supports the case for an increased accumulation of raffinose in Veyo throughout the entire vernalization period.

**Figure 7:**
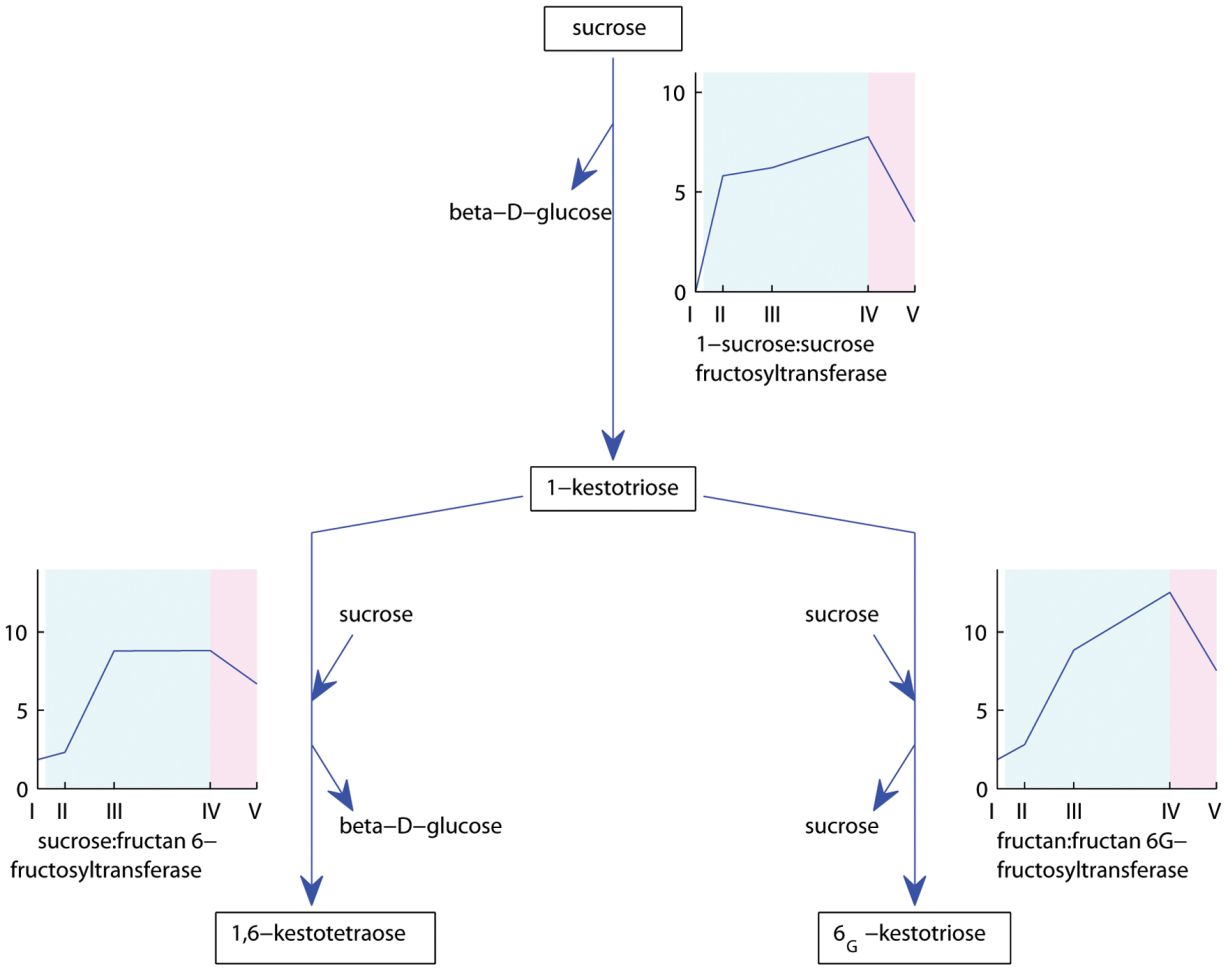
Expression profiles of Falster and Veyo transcripts involved in fructan biosynthesis. Falster transcripts are depicted in blue, Veyo transcripts in red. The *x* axis presents the sample collection time points: I =  autumn conditions, before the start of vernalization; II = 2 days of vernalization; III = 4 weeks of vernalization; IV = 9 weeks of vernalization; V = 7 days of long day conditions. The *y* axis corresponds to the variance stabilized data from DESeq. The blue background illustrates vernalization treatment, the pink background illustrates long day conditions. Figure adapted from the Plant Metabolic Pathways database (http://www.plantcyc.org/).

Transcripts with homology to enzymes involved in fructan biosynthesis, 1-sucrose:sucrose fructosyltransferase (1-SST), sucrose:fructan 6-fructosyltransferase (6-SFT), and fructan:fructan 6G-fructosyltransferase (6G-FFT), were regulated by the induction treatments only in Falster. As we have already seen, sucrose synthase transcripts were up-regulated during vernalization. Sucrose is the precursor of fructans, but has also been shown to initiate the expression of fructosyltransferases [92], [93]. It is already widely known that fructans accumulate under low temperatures in grasses adapted to cold and capable of freezing tolerance [94]–[97]. Following vernalization, fructans may represent an easily accessible energy source for the processes mediating the transition from vegetative to reproductive phase of the plant. A 1-sucrose:sucrose fructosyltransferase (1-SST) transcript showed up-regulation in leaves already at two days of vernalization, with a peak at nine weeks of vernalization (Fig. 3 cluster 5). In an initial step in fructan biosynthesis, 1-SST mediates the synthesis of 1-kestotriose from sucrose (Figure 7). The rapid up-regulation of this gene would assure thus the availability of 1-kestotriose from the early phase of cold treatment. In the next steps, the synthesis of 1,6-kestotetraose and 6_G_-kestotriose from 1-kestotriose and sucrose is mediated by 6-SFT and 6_G_-FFT, respectively. A transcript with greatest homology to 6-SFT, and another with greatest homology to 6_G_-FFT had a slower response, with a strong increase at four weeks and maintained until the end of vernalization, which could be correlated with the availability of their substrate 1-kestotriose (Fig. 3 cluster 30). All three fructosyltransferase transcripts showed a decrease in expression in leaves after the transition to long days. Similar profiles for this fructosyltransferase were previously reported in perennial ryegrass under cold treatment [97]. After an initial increase of 1-SST and 6_G_-FFT, Hissano et al. described a decrease in expression level at four days of cold treatment followed by another increase up to 30 days. This decrease could not be captured in our experiment due to the different collection time points. However, we confirmed an increased expression level of these fructosyltransferase transcripts until nine weeks of vernalization in the perennial ryegrass genotype originating from northern Europe.

**Figure 8:**
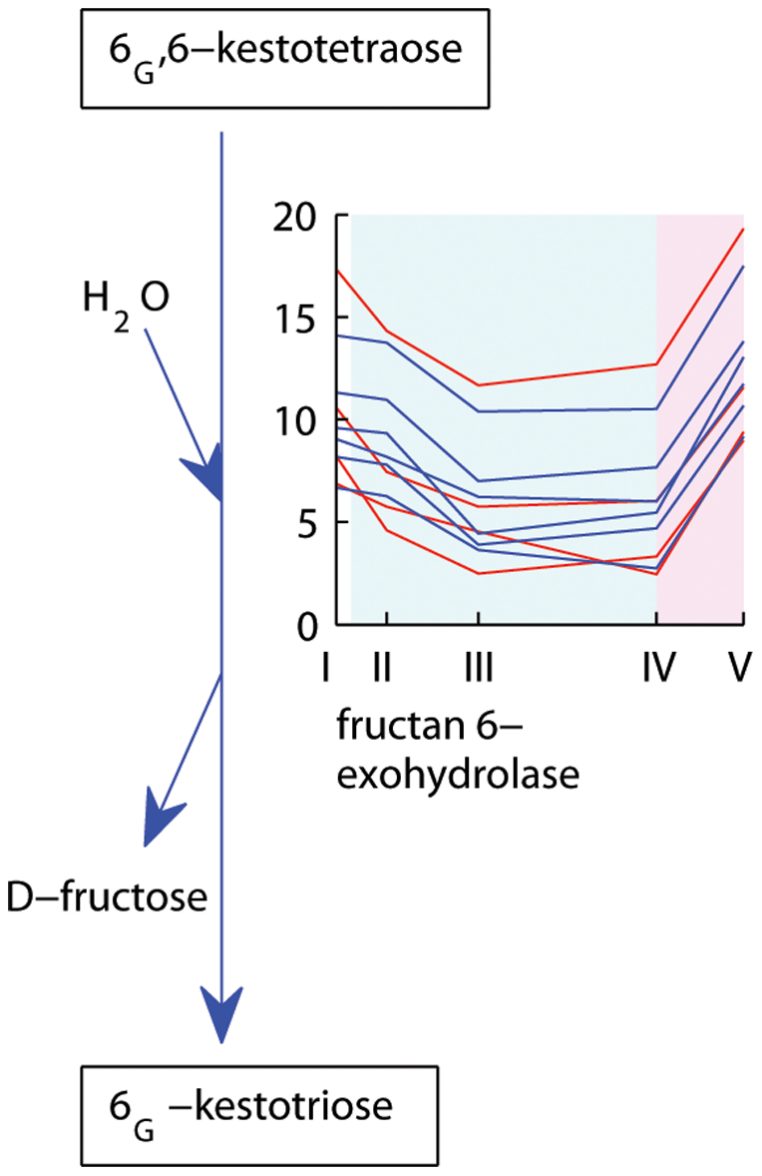
Expression profiles of Falster and Veyo transcripts involved in fructan degradation. Falster transcripts are depicted in blue, Veyo transcripts in red. The *x* axis presents the sample collection time points: I =  autumn conditions, before the start of vernalization; II = 2 days of vernalization; III = 4 weeks of vernalization; IV = 9 weeks of vernalization; V = 7 days of long day conditions. The *y* axis corresponds to the variance stabilized data from DESeq. The blue background illustrates vernalization treatment, the pink background illustrates long day conditions. Figure adapted from the Plant Metabolic Pathways database (http://www.plantcyc.org/).

Six Falster and four Veyo putative splice variants with homology to fructan exohydrolase showed differential regulation in both leaf and enriched meristem samples of the two genotypes. This is an enzyme involved in fructan degradation (Figure 8). In Falster, these transcripts were down-regulated at four and nine weeks of vernalization and were up-regulated upon transition to long days (Fig. 3 clusters 4, 15). Fructan exohydrolase transcripts displayed a similar expression profile in Veyo, with the exception of a more rapid down-regulation from the beginning of vernalization (Fig. 4 clusters 6, 9). Together with the up-regulation of fructosyltransferases, this supports an increase in fructans in both genotypes during cold. This may serve to mobilize fructan reserves to fuel new growth and the transition to reproductive growth that follows vernalization. In the enriched meristems of both genotypes, fructan exohydrolase transcripts were down-regulated at seven long days compared to their expression level at nine weeks of vernalization, suggestive of decreased fructan degradation in meristems upon transition to long days (Fig. 5 cluster 12; Fig. 6 cluster 9).

**Figure 9:**
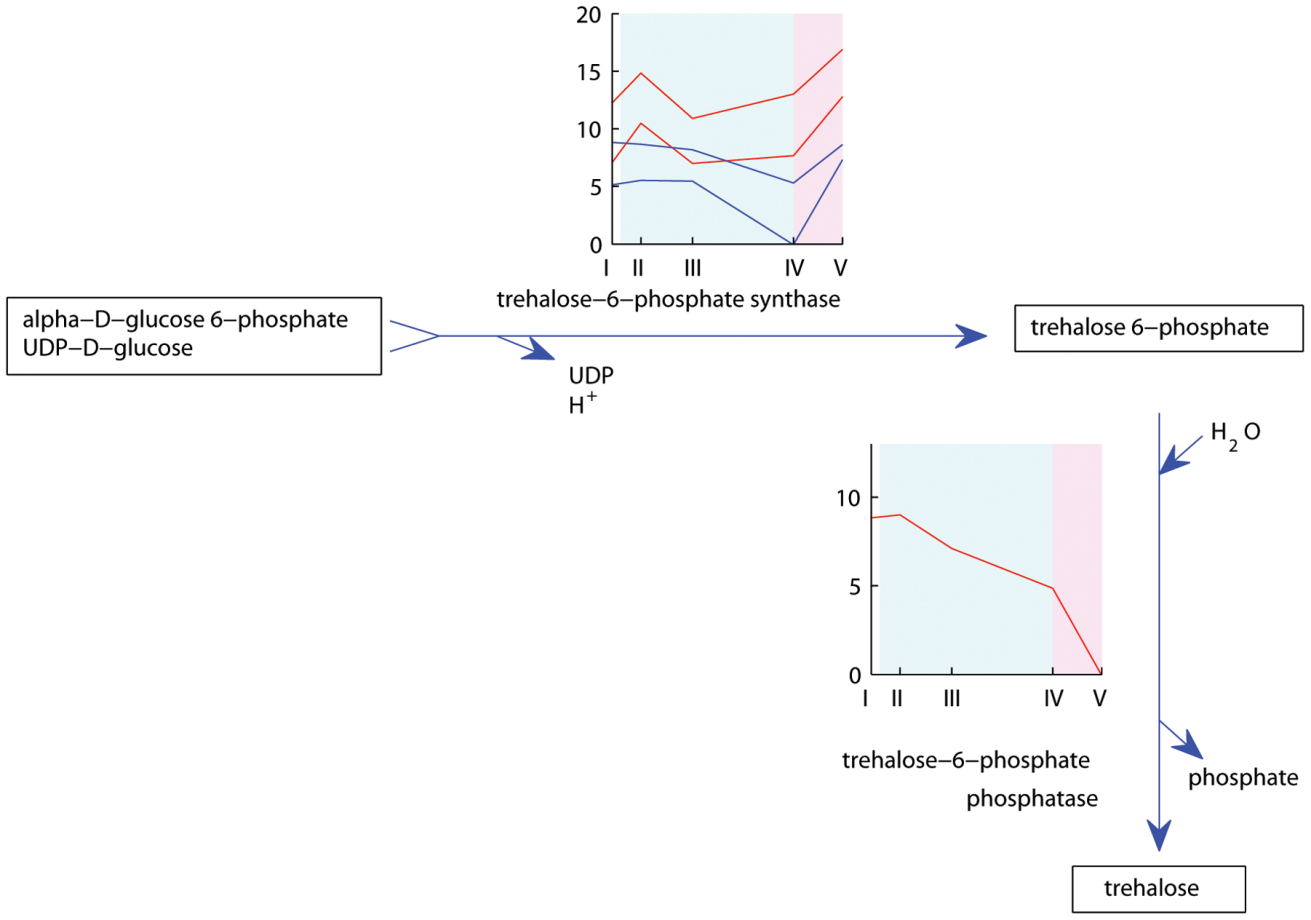
Expression profiles of Falster and Veyo transcripts involved in trehalose-6-phosphate synthesis and degradation. Falster transcripts are depicted in blue, Veyo transcripts in red. The *x* axis presents the sample collection time points: I =  autumn conditions, before the start of vernalization; II = 2 days of vernalization; III = 4 weeks of vernalization; IV = 9 weeks of vernalization; V = 7 days of long day conditions. The *y* axis corresponds to the variance stabilized data from DESeq. The blue background illustrates vernalization treatment, the pink background illustrates long day conditions. Figure adapted from the Plant Metabolic Pathways database (http://www.plantcyc.org/).

Trehalose is a disaccharide consisting of two glucose units bound by alpha, alpha-(1–>1) linkage, and it has been implicated as having a role in enhancing tolerance to abiotic stress [98]. Trehalose is synthesized when trehalose-6-phosphate, which is formed by the action of trehalose-6-phosphate synthase (TPS) on alpha-D-glucose 6-phosphate UDP-D-glucose, is degraded by treaholose-6-phosphate phosphatase (TPP) (Figure 9). Transcripts with homology to TPS and TPP were differentially regulated through floral induction. Different transcripts, annotated as TPS-like based on similarity to *Brachypodium* sequences, were differentially expressed in the leaves of the two genotypes. TPS6-like and TPS11-like transcripts were up-regulated in Veyo leaves after two days followed by a decrease during the rest of vernalization and up-regulation in long days (Fig. 4 clusters 11, 29). The TPS-like transcripts were up-regulated at seven long days in Veyo enriched meristem (Fig. 6 clusters 8, 9). In Falster leaves, TPS7-like and TPS8-like transcripts had a different profile. They were down-regulated only at nine weeks of vernalization, which is when Falster has fulfilled its vernalization requirement (Fig. 3 cluster 25). Two transcripts annotated as TPP were differentially expressed in Veyo leaf and enriched meristem. One TPP transcript was down-regulated in Veyo leaves at the end of vernalization and in long days (Fig. 4 cls 2), while another was down-regulated in enriched meristem at seven long days (Fig. 6 cluster 13). The trehalose biosynthesis pathway is particularly interesting in terms of floral induction because recently the signaling molecule TPS1 was shown to regulate the transition to flowering in *Arabidopsis* plants [99]. TPS1 is required in leaves for the induction of the florigen signal through the photoperiod flowering pathway, while in the shoot apical meristem it is connected to flowering genes from the age dependent flowering pathway [100]. Our results show up-regulation of TPS-like transcripts along with down-regulation of TPP transcripts, indicating an accumulation of trehalose-6-phosphate, promoting the transition to flowering. This is observed at seven long days in Veyo, in both leaf and enriched meristem samples. In Falster however, TPS was regulated only in leaves, where TPS7-like and TPS8-like transcripts showed down-regulation at nine weeks of vernalization, while TPP was not regulated in this genotype.

## Conclusions

RNA-Seq enabled an untargeted profiling of the transcriptome of two perennial ryegrass genotypes with contrasting vernalization requirement. Expression profiling through vernalization identified a series of transcripts with patterns suggestive of a role in floral induction. Similarities were observed between the two genotypes, as well as differences related to cold acclimation, carbohydrate metabolism, and components of the floral induction pathways. Of particular interest was the differential regulation in the two genotypes of transcripts involved in the biosynthesis and degradation of trehalose-6-phosphate, suggestive of playing a role in the floral transition not only in *Arabidopsis*, but also in grasses.

## Supporting Information

Figure S1 **The number of Falster and Veyo transcripts identified as differentially expressed within pairwise comparisons between the selected time points.**
(PNG)

Figure S2 **Differential GO term distribution between transcripts differentially expressed in the leaf samples of Falster (blue) and Veyo (red) genotypes, based on their Blast2GO annotations.** *p*<0.01.
(PNG)

Figure S3 **Differential GO term distribution between transcripts differentially expressed in the enriched meristem samples of Falster (blue) and Veyo (red) genotypes, based on their Blast2GO annotations.** *p*<0.01.
(PNG)

Figure S4 **Differential GO term distribution between the set of Falster transcripts differentially expressed between the non-inductive time point and any of the time points during primary induction (blue) compared to the whole set of differentially expressed transcripts from Falster leaf samples (red).** *p*<0.01.
(PNG)

Supplementary Material S1 **Description of the** ***K*** **-means clustering algorithm adapted to take into account the distance in time between the selected sample collection time points.**
(DOCX)

Supplementary Material S2 **RT-PCR experiment details for validation of the selected differentially expressed transcripts.**
(DOCX)

Supplementary Material S3 **Table presenting the differentially expressed Falster and Veyo transcripts with their specific BLASTx results and annotation from Blast2GO, separate for leaf and enriched meristem samples.** The table includes the cluster number in which each transcript was assigned based on the specific expression profiles presented in Fig. 3, Fig. 4, Fig. 5 and Fig. 6. The first column indicates if a transcript was found differentially expressed in both genotypes based on a common reference transcriptome (‘c’ transcript differentially expressed in both genotypes; ‘n’ transcript not present in the common reference).
(XLSX)

Supplementary Material S4 **Results of the RT-PCR validation of the RNA-Seq experiment.**
(DOCX)

Supplementary Material S5 **Individual expression profiles of the transcripts corresponding to the VRN1, GI, LHY, PRR37, CO9-LIKE, and galactinol-synthase genes observed in the leaves of Falster and Veyo genotypes.**
(DOCX)
