# Supplementary figures and images for: Vernalization Mediated Changes in the Lolium perenne Transcriptome
Source: PLoS One. 2014 Sep 16;9(9):e107365. doi: 10.1371/journal.pone.0107365 (PMC4167334; doi:10.1371/journal.pone.0107365)

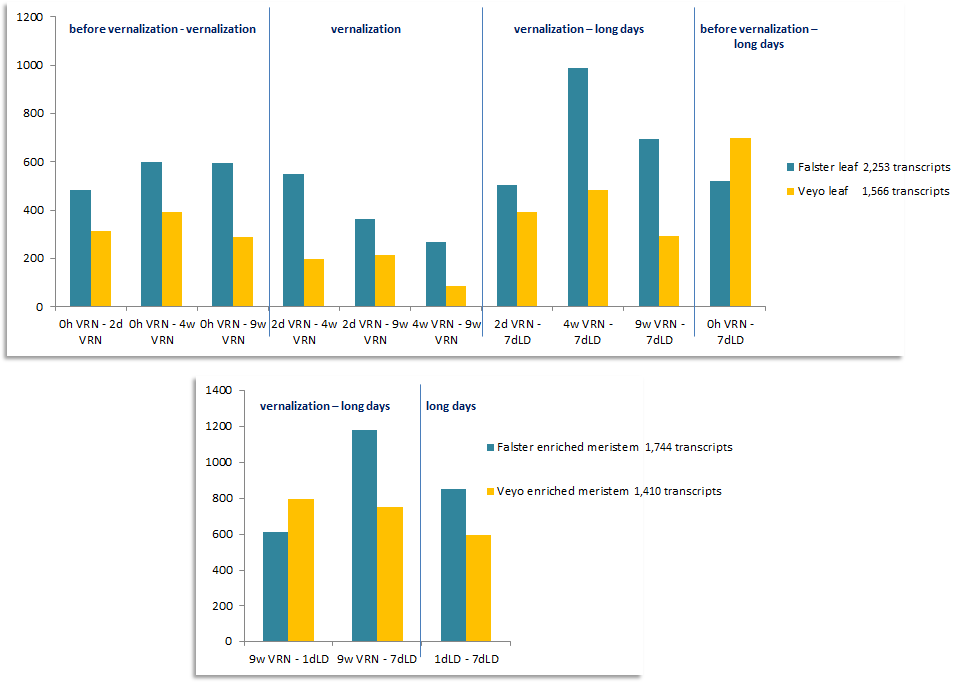

Supplement: Figure S1 — The number of Falster and Veyo transcripts identified as differentially expressed within pairwise comparisons between the selected time points. (PNG) [file pone.0107365.s001.png]

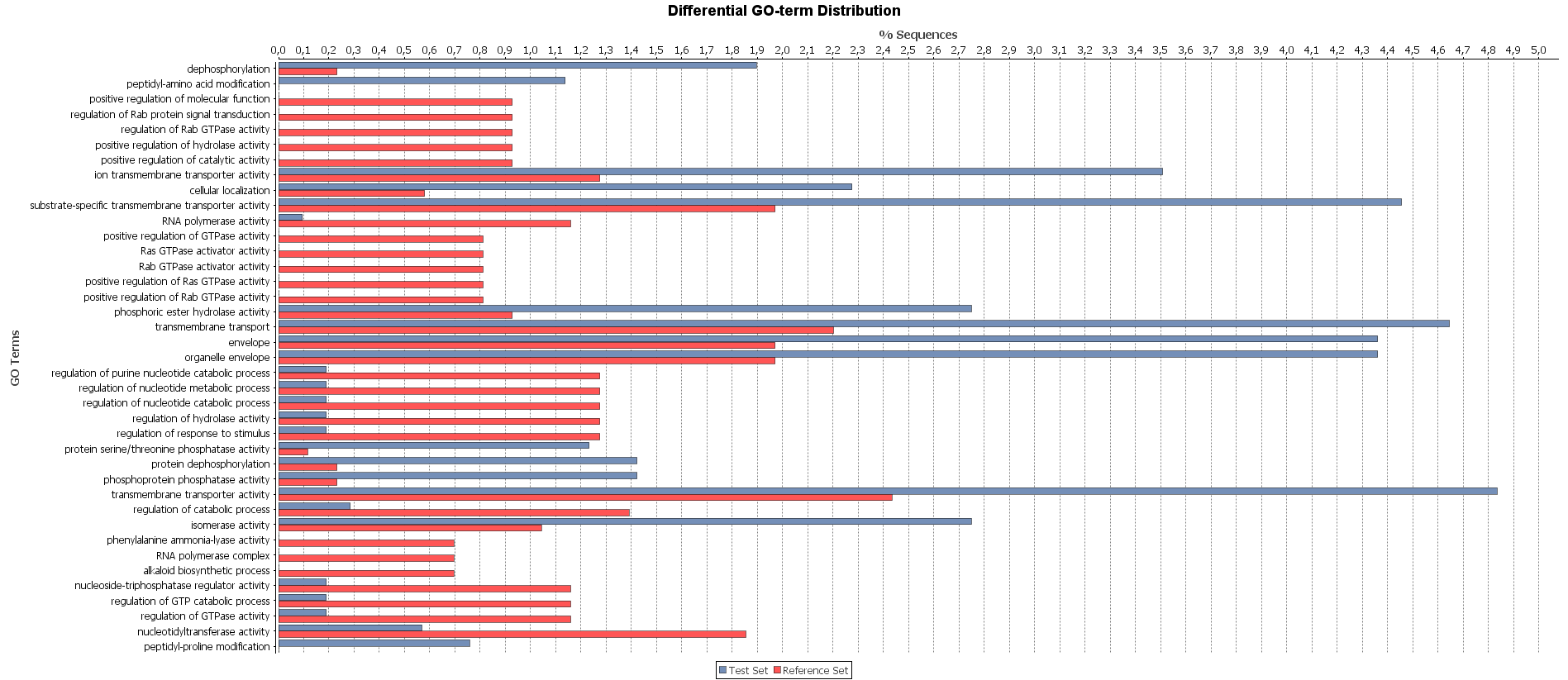

Supplement: Figure S2 — Differential GO term distribution between transcripts differentially expressed in the leaf samples of Falster (blue) and Veyo (red) genotypes, based on their Blast2GO annotations. p<0.01. (PNG) [file pone.0107365.s002.png]

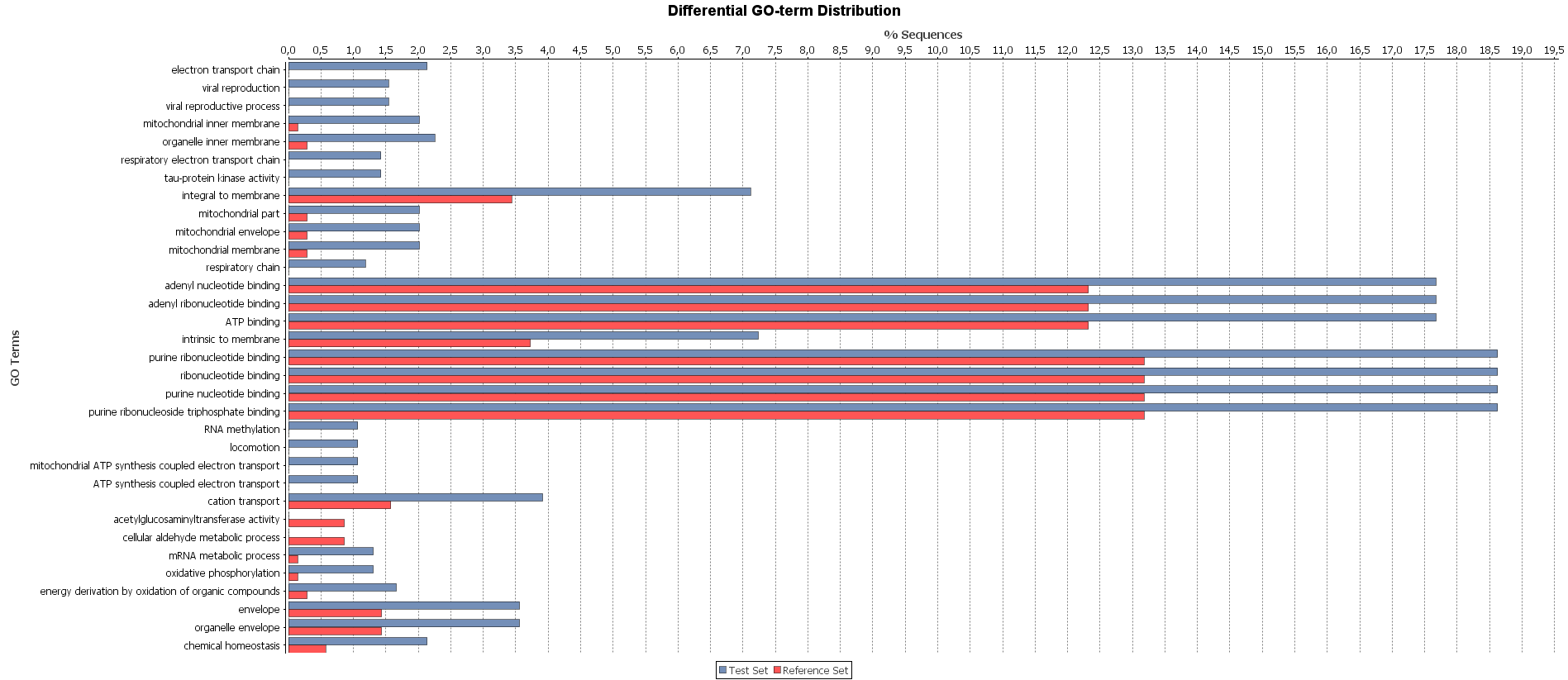

Supplement: Figure S3 — Differential GO term distribution between transcripts differentially expressed in the enriched meristem samples of Falster (blue) and Veyo (red) genotypes, based on their Blast2GO annotations. p<0.01. (PNG) [file pone.0107365.s003.png]

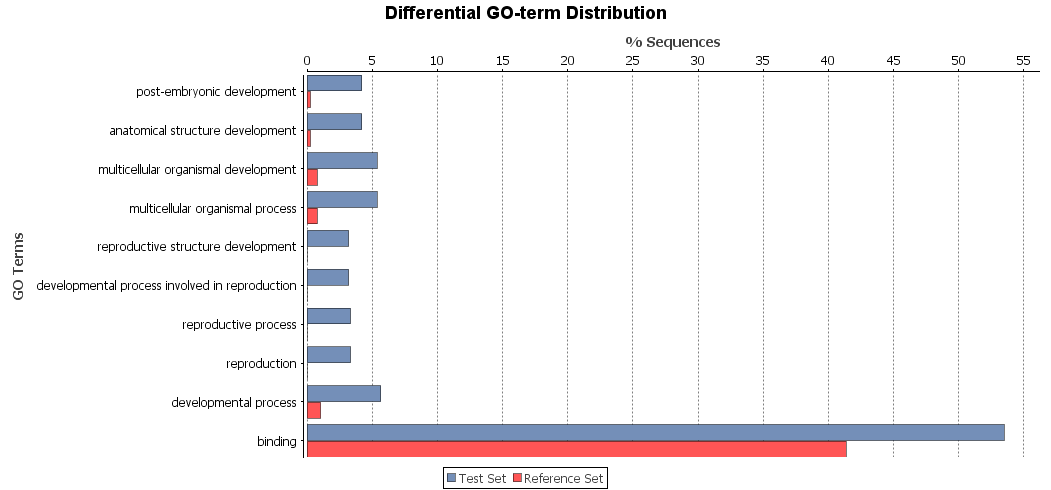

Supplement: Figure S4 — Differential GO term distribution between the set of Falster transcripts differentially expressed between the non-inductive time point and any of the time points during primary induction (blue) compared to the whole set of differentially expressed transcripts from Falster leaf samples (red). p<0.01. (PNG) [file pone.0107365.s004.png]
