# Supplementary material for: Vernalization Mediated Changes in the Lolium perenne Transcriptome
Source: PLoS One. 2014 Sep 16;9(9):e107365. doi: 10.1371/journal.pone.0107365 (PMC4167334; doi:10.1371/journal.pone.0107365)
Supplement: Supplementary Material S1 — Description of the K -means clustering algorithm adapted to take into account the distance in time between the selected sample collection time points. (DOCX) [file pone.0107365.s005.docx]

***k*-means clustering algorithm adapted to take into account the distance in time between the selected sample collection time points during primary and secondary induction.**

Typically, clustering methods do not take into account information about the relationship that may exist between the components of the experiment. In other words, typical clustering algorithms assume that each dimension characterizing a data point is equally important and the order of those dimensions does not matter. In this particular research case, we know that the five collection points are not equidistant in time but rather follow a specific schedule. It is of particular interest the fact that the “distance” in time between the first sample collection time point (T1) and the second (T2) is significantly shorter than the distance between T2 and T3, the third sample collection time point. The proposed clustering method is taking into account this distance by expanding the dimensionality of the problem. In this way, the number of columns of data increases while the expected benefit is an improved clustering result.

For the two experiments, with samples collected at five (leaf) or respectively three (enriched meristem) different moments in time, we can layout the “distance” matrix in time between the different points as follows.

Five collection time points :

| Distance | T 1* | T 2 | T 3 | T 4 | T 5* |
| --- | --- | --- | --- | --- | --- |
| T 1* | 0 | 2 days | 28 days | 63 days | 70 days |
| T 2 | 2 days | 0 | 26 days | 61 days | 68 days |
| T 3 | 28 days | 26 days | 0 | 35 days | 42 days |
| T 4 | 63 days | 61 days | 35 days | 0 | 7 days |
| T 5* | 70 days | 68 days | 42 days | 7 days | 0 |

T1= before the start of vernalization

T2= two days of vernalization

T3= four weeks of vernalization

T4= nine weeks of vernalization

T5= seven long days with higher temperatures

* indicates that this particular time point/sample could be considered independent from the others. The independence in this case comes from the fact that the environment conditions (applied treatment) are different. For instance, a plant exposed X amount of time to cold after which we examine a sample T2, then re-exposed consecutively to the same cold condition and examined again as sample T3, could intuitively be considered as being observed in two related observations. It could be argued that, whatever processes started for T2, continued for T3 (because the conditions were identical) and, therefore, T2 and T3 can be assumed to be dependent. Even if T5 comes after T1, .., T4 (because of the order in which the samples were collected), since the environmental conditions for T5 are radically different from the conditions for T1, …, T4 (warm versus cold temperatures) we can consider that the dependence between T2, T3, T4 is certainly stronger than the dependence between T5 and each of the T1, …, T4. Subsequently, we can consider that T5 is independent from T1, …, T4.

Three collection time points:

| Distance | T 1* | T 2 | T 3 |
| --- | --- | --- | --- |
| T 1* | 0 | 1 day | 7 days |
| T 2 | 1 day | 0 | 6 days |
| T 3 | 7 days | 6 days | 0 |

*T1= nine weeks of vernalization

T2= one day of long day with higher temperatures

T3= seven days of long days with higher temperatures

The proposed clustering technique augments the number of columns in the data with additional values to account for the shape of the graph. Thus, for a data set with five initial columns T1, …, T5 we add 20 more columns, four new columns for each existing column, representing the distance between the considered time point and the others. The figure below depicts an example for the sample collection time point T3:

T1

T2

T3

T4

T5

angle

One single angle is being represented in this figure. The example shows, with vertical bars, the hypothetical expression levels we might have for a particular gene reflected in the hight of each bar. The angle shown is made by the segment uniting the location on the “time” axis of the third measurement with the actual expression level of the second measurement, in a pairwise comparison between T2 and T3. Since we have five different measurements, 20 different such angles could be calculated in case of each gene in the study.

After adding the angle columns to the initial data columns, the ensemble is submitted to the k-means clustering technique. The only difference is that now, the clustering algorithm has to use 25 columns instead of five. Because most of those columns account for the shape of the gene’s variation in expression, it follows that the clustering result should group the sequences together more reliably if they have similar expression patterns. This algorithm was tested on an artificial data set, as described below.

As an example of using the proposed clustering technique, an artificial data set (Test_xD_Clustering) has been constructed containing 400 genes numbered from 1 to 400. The genes were “designed” to follow four different clusters. The first 100 genes belong to the first cluster, the next 100 to the second, and so on. A graph over imposing those genes in plots corresponding to their clusters follows:

There are only three measurements (T1, T2 and T3) and in the graph above the distance in time between measurements is the same. Running a typical clustering algorithm, we obtain the groups displayed in the next figure:

One observation that can be made is that the order of the clusters is changed from our original design, in the sense that the “green” cluster which was our cluster number 1, in the clustering results is the cluster number 2. This is a typical situation with clustering, where the order of groups is not guaranteed. Other than that, for this particular example, we did not obtain any difference. This means that all the genes that through our initial “design” where in one group, were found to belong to the same group by the clustering algorithm (nothing plus, nothing minus).

Let’s consider now that the distance in time between measurements is not equal, but rather [0, 0.1, 2.0] which would represent the moments in time when the three measurements were taken.

In this case, the two groups of graphs become:

where visually, we can no longer distinguish 4 groups (or clusters) but rather 2 (one with a descending expression profile (top figures) and the second group with an ascending profile (the bottom figures). Clearly, the clustering of such data should account for such a striking visual intuitive clustering.

Using the proposed clustering technique (augmenting the data set with shape related angles), and running the clustering algorithm with the same parameters, we obtain the clusters in the next figure. One can observe that the clusters 1,1(top left), 2,1 (bottom left), and the clusters 1,2 (top right) rigorously contain the “ascending” gene profiles while 2,2 (bottom right) contains the “descending” profiles. This is more illustrative than the previous result where data was still grouped in four different clusters in disregard of their visual appearance.

In order to better show the clusters 1,2 and 2,2, in the figure bellow, the middle measurement was represented at equal distance from the other two. This demonstrates that the “green” and “red” clusters where assigned to the same group because when the distance between the first and second measurement is very small, the two clusters are undistinguishable.

This experiment demonstrates that our proposed clustering algorithm which includes an augmented number of dimensions as described, is better at preserving the “shape” or the pattern of the expression profiles. In other words, genes displaying similar expression profiles will have a better chance to end up in the same cluster when comparing with typical clustering algorithms. When the number of expected clusters was reduced to 2 in this artificial experiment, all “upward” patterns ended up in one cluster and the “downward” ones in the other.

Test_xD_Clustering data set:

Gene T1 T2 T3

1 1 1.2 0

2 1.010991 1.052523 0.083176

3 0.85566 1.125685 -0.12206

4 1.107578 1.107914 0.137112

5 1.06745 1.087589 -0.08153

6 0.900823 1.266187 -0.06922

7 0.909491 1.070527 0.060168

8 1.052812 1.213041 0.025004

9 0.962158 1.09907 -0.14482

10 0.994808 1.171561 0.135192

11 0.970868 1.281696 0.036621

12 0.953913 1.28133 0.130609

13 0.946585 1.077 -0.03695

14 1.063408 1.276548 0.118322

15 0.99693 1.195488 0.109762

16 1.037348 1.163942 -0.14209

17 1.132536 1.335518 -0.03358

18 1.05406 1.193343 0.093761

19 1.097552 1.180098 -0.09146

20 1.016756 1.188755 0.043919

21 1.088833 1.333041 0.024376

22 1.083831 1.207486 -0.02116

23 1.088253 1.171754 -0.02334

24 0.906815 1.288086 0.112434

25 0.901038 1.161956 -0.02082

26 0.875483 1.136901 0.000335

27 0.863722 1.121153 -0.00582

28 0.918924 1.31939 -0.07551

29 1.144784 1.100647 -0.12925

30 0.900908 1.259583 -0.04812

31 1.062234 1.192415 -0.05958

32 1.029737 1.301861 -0.12045

33 0.994889 1.141119 0.050078

34 0.866012 1.231542 -0.14842

35 1.07332 1.210777 0.103627

36 1.031103 1.235147 -0.04029

37 0.859122 1.247079 0.108715

38 0.855111 1.102581 -0.08195

39 0.936792 1.309976 -0.07101

40 1.060042 1.292387 0.12596

41 1.056383 1.155345 0.057904

42 0.999894 1.051445 0.066711

43 1.001946 1.173381 0.016047

44 1.077356 1.276733 0.089418

45 0.873438 1.057939 -0.05481

46 0.917714 1.10481 -0.14855

47 1.027731 1.18487 0.009732

48 0.984486 1.077706 -0.10157

49 0.909479 1.088422 0.004598

50 1.111054 1.287805 -0.04066

51 0.942665 1.267074 -0.06873

52 0.901171 1.167462 -0.08224

53 0.861351 1.15469 0.031465

54 1.027266 1.210168 -0.01376

55 0.871626 1.258624 0.087231

56 1.10873 1.163008 0.056085

57 0.983417 1.324244 0.046868

58 1.096258 1.286663 -0.13514

59 0.864381 1.051023 -0.09479

60 1.027706 1.257432 0.061999

61 0.88412 1.091385 0.1342

62 0.885977 1.20771 0.006298

63 0.937408 1.226498 -0.05486

64 0.897238 1.100941 0.035803

65 0.93425 1.077601 -0.13526

66 0.928557 1.244156 0.149424

67 0.979143 1.280676 0.141449

68 1.108016 1.326768 0.075572

69 0.996598 1.251276 -0.02252

70 1.139688 1.061371 -0.13497

71 1.030965 1.159396 -0.12474

72 1.128296 1.256359 0.092413

73 1.133036 1.080532 0.0123

74 1.08353 1.079567 0.009875

75 0.897342 1.063952 -0.00379

76 1.070531 1.075113 -0.10632

77 1.059675 1.2294 -0.13481

78 1.148483 1.285841 -0.05152

79 1.112745 1.084854 -0.02573

80 1.062377 1.102182 -0.07594

81 0.959399 1.309974 -0.08747

82 1.117835 1.189353 -0.09233

83 1.017587 1.058533 -0.07198

84 0.981507 1.186013 -0.00484

85 0.916903 1.215825 -0.07381

86 1.077086 1.182062 0.099116

87 0.970356 1.225282 -0.02475

88 0.993247 1.103549 -0.06836

89 0.861489 1.342228 0.077243

90 0.989294 1.190797 -0.09277

91 0.901522 1.15599 -0.06005

92 0.904645 1.256511 0.079096

93 1.0037 1.181346 -0.1121

94 1.062263 1.111893 0.086234

95 1.044986 1.306247 0.105014

96 0.870325 1.248919 0.099398

97 1.070796 1.322231 -0.03338

98 0.946667 1.193754 0.079856

99 1.080559 1.110236 0.068068

100 0.898295 1.139345 -0.09093

101 1.4 1.2 0

102 1.272116 1.059657 0.066343

103 1.344591 1.108923 -0.13404

104 1.257088 1.293884 -0.02767

105 1.478544 1.325152 0.113875

106 1.495601 1.246814 -0.02728

107 1.277754 1.324554 -0.00385

108 1.421699 1.145466 0.108538

109 1.471701 1.167797 -0.06546

110 1.359403 1.065303 -0.11195

111 1.283497 1.217351 0.13576

112 1.311561 1.066157 0.08104

113 1.459544 1.264067 0.132783

114 1.51388 1.091105 0.033286

115 1.461039 1.249199 -0.12797

116 1.325283 1.052288 0.123849

117 1.270591 1.262142 -0.09582

118 1.362403 1.156643 0.083351

119 1.410761 1.22074 -0.0797

120 1.46117 1.268866 -0.13429

121 1.472174 1.31046 0.052123

122 1.467467 1.18936 -0.07694

123 1.334317 1.118424 -0.14064

124 1.538633 1.259288 -0.1482

125 1.307628 1.281001 0.149841

126 1.273966 1.173261 -0.1362

127 1.425547 1.341305 -0.0339

128 1.387758 1.283187 -0.03333

129 1.434826 1.191853 0.101958

130 1.42007 1.097585 -0.10593

131 1.455787 1.171893 -0.07788

132 1.500235 1.230556 -0.08169

133 1.349784 1.177023 -0.03301

134 1.54536 1.243896 0.071107

135 1.491311 1.081113 -0.07664

136 1.491559 1.242913 -0.09121

137 1.376995 1.327148 0.026984

138 1.31004 1.33128 0.143548

139 1.349982 1.131089 0.124082

140 1.486261 1.183511 -0.0189

141 1.270437 1.286589 -0.11997

142 1.282035 1.162001 -0.00288

143 1.37519 1.067527 -0.06381

144 1.256947 1.160528 0.050702

145 1.456495 1.303641 -0.05388

146 1.403624 1.264381 -0.12238

147 1.278637 1.168847 -0.01721

148 1.499764 1.141676 0.146789

149 1.512632 1.202848 -0.11313

150 1.345206 1.197703 -0.07739

151 1.52953 1.112528 0.124161

152 1.447346 1.282593 0.127044

153 1.391174 1.308055 0.070607

154 1.465387 1.290081 -0.1333

155 1.296254 1.144398 0.08184

156 1.419715 1.268811 0.054115

157 1.291895 1.132862 -0.04423

158 1.363314 1.193964 0.022857

159 1.330442 1.304647 -0.05557

160 1.300496 1.349406 -0.05741

161 1.391579 1.098559 0.014269

162 1.29336 1.066957 0.08831

163 1.331945 1.076312 0.011399

164 1.518613 1.212884 0.051562

165 1.310032 1.097949 0.05245

166 1.454457 1.296101 0.111323

167 1.479196 1.296822 0.013461

168 1.453601 1.149816 -0.14923

169 1.545718 1.085768 0.065545

170 1.318019 1.243372 -0.11888

171 1.49116 1.263839 -0.13979

172 1.453951 1.336494 0.081811

173 1.48924 1.081299 0.086129

174 1.359707 1.346389 0.075571

175 1.452556 1.104527 -0.04613

176 1.512934 1.341527 -0.12678

177 1.314007 1.263621 -0.07207

178 1.498801 1.232014 0.036378

179 1.499527 1.058068 0.111213

180 1.539118 1.288118 -0.00223

181 1.288312 1.160369 -4.7E-05

182 1.267098 1.240278 0.009125

183 1.274528 1.164725 0.037572

184 1.398624 1.30281 0.030098

185 1.441965 1.216311 0.045978

186 1.425367 1.327343 -0.02854

187 1.499483 1.344082 0.011366

188 1.528346 1.315818 0.051622

189 1.537143 1.324695 -0.073

190 1.466797 1.242115 -0.05532

191 1.368683 1.061418 0.011264

192 1.450468 1.237231 0.064858

193 1.288305 1.161596 0.129303

194 1.349727 1.136112 0.004083

195 1.427157 1.168274 0.064979

196 1.276294 1.332855 0.032343

197 1.383629 1.271454 0.104846

198 1.392948 1.052972 0.007368

199 1.521206 1.319043 -0.01563

200 1.339167 1.230792 0.136928

201 0 0.2 1

202 0.067912 0.105549 1.120123

203 -0.0698 0.139221 0.894762

204 0.071792 0.066668 1.123323

205 0.089658 0.140116 0.910015

206 -0.04822 0.279725 0.878229

207 -0.14295 0.111279 1.032324

208 0.13737 0.190573 0.892264

209 -0.02787 0.292349 0.967684

210 -0.1456 0.26904 0.954718

211 0.038048 0.264684 1.141008

212 0.005747 0.341975 1.123981

213 0.033963 0.227696 1.009309

214 -0.10958 0.11397 1.018722

215 0.010635 0.244784 0.917596

216 -0.04025 0.348378 0.891338

217 0.031364 0.308482 0.914489

218 0.084396 0.344812 0.864013

219 0.144935 0.211926 0.883993

220 0.008129 0.264945 0.865975

221 -0.04701 0.063596 1.011057

222 -0.05274 0.065241 0.905434

223 -0.00579 0.322309 0.918802

224 -0.00986 0.075183 0.941884

225 0.122921 0.053223 1.005843

226 0.122959 0.253234 0.853956

227 0.14364 0.305326 1.073484

228 0.105721 0.090257 1.110813

229 -0.11112 0.19917 1.131011

230 0.017444 0.127411 0.935615

231 -0.06279 0.090428 1.094985

232 0.080136 0.238198 1.009533

233 -0.0396 0.087662 0.951092

234 -0.02577 0.273383 0.999331

235 0.118982 0.09987 0.973261

236 0.130478 0.324838 0.958563

237 0.013644 0.118393 1.12998

238 0.076733 0.316168 1.006485

239 -0.11333 0.131373 0.87238

240 0.088974 0.146054 0.908579

241 0.031481 0.160887 0.918972

242 -0.04568 0.256471 0.988574

243 0.077314 0.301728 1.109824

244 -0.13615 0.052066 1.004216

245 -0.13959 0.055435 0.957602

246 0.072244 0.157484 0.954554

247 -0.12496 0.171266 0.933622

248 0.117475 0.305378 0.863699

249 0.136713 0.092003 1.033576

250 0.009981 0.074589 0.905903

251 0.085144 0.226824 1.055314

252 0.037525 0.184225 0.892524

253 -0.09298 0.3181 1.00163

254 -0.12339 0.235759 0.91561

255 -0.0399 0.193806 1.001954

256 0.032843 0.156821 0.979059

257 -0.05744 0.300988 0.957391

258 -0.11136 0.191851 0.932508

259 -0.13282 0.345843 1.090517

260 -0.14727 0.328687 0.933606

261 0.013403 0.146815 0.919918

262 0.099992 0.165216 0.924786

263 0.012955 0.158336 1.141459

264 -0.02591 0.218797 1.122973

265 0.057058 0.178613 1.008834

266 0.126807 0.253858 0.986676

267 -0.1007 0.173125 1.039988

268 0.121207 0.181307 0.858893

269 -0.0752 0.217337 1.064606

270 0.049082 0.166009 1.021482

271 0.106585 0.329114 0.941752

272 -0.01468 0.062564 0.937049

273 -0.14923 0.275193 1.115442

274 0.018516 0.302867 1.075781

275 -0.04588 0.189194 0.990455

276 -0.0975 0.244121 0.890882

277 0.084769 0.147523 1.013537

278 0.085576 0.181245 0.942188

279 0.055905 0.155567 0.952909

280 0.135727 0.183266 1.126196

281 -0.12429 0.211233 0.922867

282 -0.053 0.162258 1.134169

283 -0.0058 0.189073 0.980244

284 -0.04564 0.155419 0.893144

285 -0.08705 0.2868 1.121569

286 -0.01468 0.316576 1.094466

287 0.05759 0.236654 1.109393

288 0.143304 0.245309 0.900607

289 -0.11797 0.203351 0.950743

290 0.080683 0.154768 1.041925

291 -0.12174 0.348942 0.885369

292 0.143607 0.323514 0.997655

293 0.029445 0.343428 0.953039

294 -0.11788 0.339004 1.084031

295 -0.14582 0.291189 0.873227

296 -0.04468 0.246535 1.008504

297 -0.12367 0.096531 0.944884

298 -0.13838 0.20717 1.088873

299 -0.02183 0.288791 0.995787

300 -0.00678 0.119246 0.994626

301 0.4 0.2 1

302 0.303061 0.053759 0.858232

303 0.44758 0.234558 1.067907

304 0.438032 0.199019 1.129214

305 0.386776 0.171451 1.146893

306 0.503458 0.25679 1.129005

307 0.548049 0.190181 0.890246

308 0.253632 0.305834 1.012717

309 0.371685 0.159632 1.074433

310 0.43169 0.309157 1.102048

311 0.520573 0.202775 0.858537

312 0.264174 0.302982 0.942472

313 0.355715 0.14259 1.106669

314 0.530139 0.11275 0.912697

315 0.342518 0.136924 0.936617

316 0.483264 0.18444 0.882935

317 0.317252 0.119197 1.090461

318 0.503467 0.341683 0.966433

319 0.374723 0.276099 0.879229

320 0.339288 0.235364 0.985316

321 0.526648 0.085626 1.034546

322 0.528648 0.105898 0.986093

323 0.480503 0.304158 1.023843

324 0.288543 0.226282 1.087112

325 0.531967 0.237157 1.122791

326 0.313248 0.150213 0.861475

327 0.445841 0.203928 1.143816

328 0.519283 0.198244 0.855341

329 0.495252 0.07125 0.895531

330 0.402501 0.137251 1.061281

331 0.485378 0.100358 1.092918

332 0.413495 0.180994 0.918547

333 0.278512 0.344548 1.039649

334 0.526589 0.338568 1.080843

335 0.517584 0.216346 1.054871

336 0.411361 0.296726 1.061458

337 0.32869 0.276029 1.13778

338 0.335671 0.053411 0.889004

339 0.519441 0.208825 0.965526

340 0.274096 0.057007 1.027622

341 0.337575 0.238061 0.903534

342 0.419704 0.290722 0.934678

343 0.39266 0.292918 1.050231

344 0.538509 0.256893 0.975959

345 0.317377 0.082386 1.044436

346 0.293609 0.054408 1.033772

347 0.337055 0.294403 1.032514

348 0.332617 0.263587 1.0585

349 0.36817 0.17672 0.910302

350 0.4248 0.21622 0.907342

351 0.542627 0.067475 1.146243

352 0.32947 0.290099 1.023255

353 0.47817 0.31518 1.056305

354 0.311586 0.240753 0.85869

355 0.403313 0.140551 1.027914

356 0.331976 0.128466 1.127678

357 0.532548 0.312475 1.048033

358 0.440263 0.209237 1.051314

359 0.311789 0.099921 0.891951

360 0.253204 0.222327 0.999475

361 0.511382 0.077717 1.054293

362 0.416772 0.291263 0.947632

363 0.448983 0.220235 0.884205

364 0.34094 0.07428 0.982686

365 0.395913 0.164924 0.890845

366 0.547922 0.295238 0.978829

367 0.437806 0.14392 1.068125

368 0.348093 0.229535 0.997967

369 0.501983 0.302495 0.887729

370 0.321704 0.149392 0.853941

371 0.34368 0.324883 0.965257

372 0.271984 0.209393 1.106511

373 0.451626 0.108421 1.015266

374 0.451914 0.345924 1.087642

375 0.414708 0.083274 1.017671

376 0.300986 0.255935 0.910634

377 0.526587 0.331499 0.991855

378 0.390241 0.342759 0.970927

379 0.516802 0.23538 1.017796

380 0.41773 0.316117 1.129598

381 0.394398 0.298251 1.0643

382 0.521065 0.077331 0.863618

383 0.397532 0.208047 0.918702

384 0.2867 0.114222 1.108977

385 0.54784 0.067552 1.019323

386 0.354129 0.117206 1.100894

387 0.452142 0.089535 1.027387

388 0.482564 0.065652 0.89473

389 0.334885 0.099517 1.002838

390 0.402775 0.271475 1.103042

391 0.358041 0.239263 1.076931

392 0.291202 0.128007 0.867336

393 0.471938 0.333272 1.079839

394 0.437793 0.149652 0.87328

395 0.30973 0.259726 1.071573

396 0.422969 0.065986 1.119624

397 0.475298 0.324056 0.858831

398 0.280393 0.050607 0.960442

399 0.466998 0.098433 0.978439

400 0.294815 0.266982 1.118789
