# Supplementary material for: Vernalization Mediated Changes in the Lolium perenne Transcriptome
Source: PLoS One. 2014 Sep 16;9(9):e107365. doi: 10.1371/journal.pone.0107365 (PMC4167334; doi:10.1371/journal.pone.0107365)
Supplement: Supplementary Material S2 — RT-PCR experiment details for validation of the selected differentially expressed transcripts. (DOCX) [file pone.0107365.s006.docx]

**RT-PCR validation**

For first-strand synthesis, 550 ng of total RNA was incubated for 5 minutes at 65°C with 1 µl of random nanomer primers, and sterile, distilled water, to add up to a total volume of 18,4 µl. The samples were chilled on ice for one minute, and briefly centrifuged. A master mix consisting of 6 µl 5x FS buffer, 3 µl 0.1M DTT, 1 µl SuperScript II (all supplied with the SuperScript II Reverse Trnascriptase Kit), 1 µl RNAsin (Promega), 0.6 µl 25 nM dNTPs, with a total volume of 11.6 µl was added to each sample, followed by a one hour incubation at 42°C, and 10 minutes incubation at 70°C. After a brief centrifugation, 170 µl of sterile, distilled water was added to each sample, resulting in a total cDNA sample volume of 200 µl.

Each RT-PCR amplification was set up in a total reaction volume of 10 µl, consisting of 5 µl of SYBRGreen PCR Master Mix, Life Technologies (Applied Biosciences), 1 µl 10 mM forward - reverse primer mix, and 4 µl of cDNA. The reactions were run on the ViiA7 Real-Time PCR System Instrument (Life Technologies, Applied Biosciences), as follows: 2 minutes at 50°C, followed by 10 minutes at 95°C, 40 cycles with 15 seconds at 95°C and 1 minute at 60°C (59°C or 64°C respectively, depending on the optimal annealing temperature of the primers). The melting curves were analyzed for each reaction to ensure the specificity of the amplification.

Supplementary Table 1. Primer sequences designed for RT-PCR validation of differentially expressed transcripts. V1049 corresponds to the house keeping (Hk) gene YT 521-B-like protein family protein; V154 corresponds to the house keeping gene eukaryotic elongation factor alpha.

| primer | primer sequence | annealing temperature ˚C | house keeping gene |
| --- | --- | --- | --- |
| veyo_1049_c0_seq2_F | TTTGCTAGCTCAAAGGCATCTC | 64 | Hkgene (V1049) |
| veyo_1049_c0_seq2_R | GAAGTTGGGAATCCGTCCTC |  |  |
| veyo_154_c0_seq1_F | GGCAAGTTCCCAAACACACT | 60 | Hkgene (V154) |
| veyo_154_c0_seq1_R | CAAGAGCGTGGAGAAGAAGG |  |  |
| falster_10241_c0_seq2_F | CACAATCAAGGTGGTCATCGT | 60 | V154 |
| falster_10241_c0_seq2_R | ATCCCAGAACCATGGTGAGA |  |  |
| falster_12737_c0_seq1_F | CTACATCGAGGAAGGGAAGCT | 64 | V1049 |
| falster_12737_c0_seq1_R | TGGGGTTATGCACACTCTGA |  |  |
| falster_14360_c0_seq1_F | GACCGCCTGGACTACTTCTG | 60 | V154 |
| falster_14360_c0_seq1_R | ACGAGGGTGTTGTCGAAGAG |  |  |
| falster_14756_c0_seq4_F | TCTGGAGGCACCTGTACAGTAAC | 60 | V154 |
| falster_14756_c0_seq4_R | CTCCACAGCATCAACAGCAG |  |  |
| falster_14982_c0_seq1_F | TTACCAGGTTGAGGGCGC | 60 | V154 |
| falster_14982_c0_seq1_R | CGAGTTGACGCCCAGATTAT |  |  |
| falster_16661_c0_seq1_F | TTGCTCTCCCTTGTACACGAA | 60 | V154 |
| falster_16661_c0_seq1_R | TCAGGAGGTCGTCTCTGAGG |  |  |
| falster_19713_c0_seq1_F | TCCCCAGAGGCAGATACATG | 60 | V154 |
| falster_19713_c0_seq1_R | GCAGCTCTTCTCTCCGTCAG |  |  |
| falster_22221_c0_seq1_F | TCTGACTCCGATACACCAAATG | 60 | V154 |
| falster_22221_c0_seq1_R | TGCACGTGAGTGCATCATTA |  |  |
| falster_24176_c0_seq3_F | GTACTCACTCCTGCACGGACA | 60 | V154 |
| falster_24176_c0_seq3_R | CTGACCACCATCAGCTACCC |  |  |
| falster_2478_c0_seq1_F | TAGCTTGGCGAAGGCCAT | 64 | V1049 |
| falster_2478_c0_seq1_R | CGAGACAGAGTCCCCAAGAA |  |  |
| falster_26638_c0_seq7_F | CTTACAGGAAGGGGAAAATGAG | 60 | V154 |
| falster_26638_c0_seq7_R | ACTAAAACCAATATGCCCAGCA |  |  |
| falster_3174_c0_seq2_F | TGCTGCATCAAGAAGCAAGT | 60 | V154 |
| falster_3174_c0_seq2_R | CAAAGGTGGCCTAGATGGAG |  |  |
| falster_5842_c0_seq1_F | GATGTTCGTCAGGGCAGTACT | 60 | V154 |
| falster_5842_c0_seq1_R | CAGCTGAGGTATCCAATTCCA |  |  |
| falster_614_c0_seq1_F | AGGAGGAAATGGACATGGTG | 60 | V154 |
| falster_614_c0_seq1_R | AGCTCACTCCCCTGTGTCAT |  |  |
| falster_6864_c0_seq5_F | GCTATGTCAGGCTTGAGAACTG | 59 | V154 |
| falster_6864_c0_seq5_R | GGCATGGCATGTATAATTGTTG |  |  |
| falster_7423_c0_seq2_F | TCAACGCCAGGGAGTATTAGTT | 59 | V154 |
| falster_7423_c0_seq2_R | CCAGAGTTTCGGTGACCATT |  |  |
| falster_8322_c0_seq2_F | CGAAGATCGGCTTGTTATCATC | 60 | V154 |
| falster_8322_c0_seq2_R | GGACTAGGGCAGTGCAGAAG |  |  |
| falster_8633_c0_seq4_F | AAACATCATGCAGGGAATCG | 60 | V154 |
| falster_8633_c0_seq4_R | ATAGCCCCGAACGACATCT |  |  |
| falster_9861_c0_seq3_F | ACACCTACAGGGCGCTGAT | 59 | V154 |
| falster_9861_c0_seq3_R | CTGGTTAAGCAGCCAGGAGA |  |  |
| veyo_10022_c0_seq1_F | CGCAAGCAGGCTTATACGAT | 60 | V154 |
| veyo_10022_c0_seq1_R | TGTTCTTCGCAAGCATTGTC |  |  |
| veyo_11872_c0_seq9_F | GGAGGATTGGCCTTTAAGGTAA | 60 | V154 |
| veyo_11872_c0_seq9_R | GCACAGAAAGAGCTGCAGAAG |  |  |
| veyo_11959_c0_seq2_F | TCCCTCTCCCTTCCGTTG | 60 | V154 |
| veyo_11959_c0_seq2_R | CCCACAAGACCCATTTATGC |  |  |
| veyo_12185_c0_seq6_F | TCTGAGTTTCCTGAGCAAGGG | 64 | V1049 |
| veyo_12185_c0_seq6_R | GGAAGATTTAGGAGGAGGCTTG |  |  |
| veyo_14961_c0_seq1_F | AGCCACATTCCTGGCTAGTG | 60 | V154 |
| veyo_14961_c0_seq1_R | CCGCAGGAAAACAAAATAGG |  |  |
| veyo_17835_c0_seq1_F | GCCTATCACCTGCATCGAGA | 60 | V154 |
| veyo_17835_c0_seq1_R | TTGTTTCCTTGCTCCTCTGG |  |  |
| veyo_21389_c0_seq5_F | CGACCACCAGATATGCTCTTG | 60 | V154 |
| veyo_21389_c0_seq5_R | CGAACTCTCTCATGGTGCAG |  |  |
| veyo_22201_c0_seq1_F | TGTAGTCGATCTGCACCCTG | 60 | V154 |
| veyo_22201_c0_seq1_R | GAGAAGACGCCCCACATCTA |  |  |
| veyo_27339_c0_seq2_F | AGGAGTGCCTTGCCCACA | 60 | V154 |
| veyo_27339_c0_seq2_R | ACGGAAGCTGAACTTGAGGA |  |  |
| veyo_28401_c0_seq1_F | TTCATCGCTGGGTTCAAGG | 60 | V154 |
| veyo_28401_c0_seq1_R | CCGTAGAAGAAGGCCACAAC |  |  |
| veyo_32335_c0_seq1_F | TTTCCATATGGCGTGAATGAC | 60 | V154 |
| veyo_32335_c0_seq1_R | ACAGTCAGCACCCTGGATTC |  |  |
| veyo_3401_c0_seq1_F | GTACCATCACCATCAAAAGAAGC | 60 | V154 |
| veyo_3401_c0_seq1_R | GAGATCCCGGGGAGTACG |  |  |
| veyo_4137_c0_seq2_F | GGATGAAATCAGACAAATGTCG | 60 | V154 |
| veyo_4137_c0_seq2_R | CGAGCTCAAGGTGACCAAA |  |  |
| veyo_4845_c0_seq1_F | CATCCACTTTGCTGCCTGA | 60 | V154 |
| veyo_4845_c0_seq1_R | AGGAGGAAACTGGGGTTGAC |  |  |
| veyo_5438_c0_seq1_F | CCAACCAGATCCTGTGGTATG | 60 | V154 |
| veyo_5438_c0_seq1_R | GAGGAAAACGTGCCCATAGA |  |  |
| veyo_7121_c0_seq2_F | AGGAAGCCAAGGTTAACCAAG | 60 | V154 |
| veyo_7121_c0_seq2_R | GGACATGTAATGCCCCTGAT |  |  |
| veyo_9357_c0_seq1_F | CGGAACTGCCGATTCCTA | 60 | V154 |
| veyo_9357_c0_seq1_R | TGGAATGAGCCTTAGCCTGT |  |  |
| veyo_9648_c0_seq3_F | CTTCAGCTGCCTTTCCTCTCT | 60 | V154 |
| veyo_9648_c0_seq3_R | GACCTGGGGGAAAAACAATC |  |  |
| veyo_9750_c0_seq3_F | GTCCAACCGCTGAATAGCAA | 60 | V154 |
| veyo_9750_c0_seq3_R | GGCCTGAAAAGGTTCTGGTAG |  |  |
