# Supplementary material for: Vernalization Mediated Changes in the Lolium perenne Transcriptome
Source: PLoS One. 2014 Sep 16;9(9):e107365. doi: 10.1371/journal.pone.0107365 (PMC4167334; doi:10.1371/journal.pone.0107365)
Supplement: Supplementary Material S4 — Results of the RT-PCR validation of the RNA-Seq experiment. (DOCX) [file pone.0107365.s008.docx]

**RT-PCR experiment for the validation of the transcripts identified as differentially expressed**

**Table presenting the results of the RT-PCR validation of 38 randomly selected transcripts.** T1…T5 represent the sample collection time points (T1 = before the start of vernalization;T2 = two days of vernalization; T3 = four weeks of vernalization; T4 = nine weeks of vernalization; T5 = seven long days). The column marked with ‘*’ refers to the total number of pairwise comparisons in which the transcript was detected as differentially expressed using the DESeq software. The green background marks transcripts for which the RT-PCR experiment showed significant changes in case of all the pairwise comparisons in which the transcript was detected as differentially expressed using DESeq (significance threshold p<0.05). The maroon color background marks transcripts for which none of the changes was confirmed as significant through the RT-PCR experiment, while the white background marks transcripts with at least one change confirmed as significant. The ‘Cluster no.’ column is related to Figure 3 for transcripts originating from Falster genotype (transcript name starting with ‘f’) and Figure 4 for Veyo transcripts (transcript name starting with ‘v’).

| **Transcript ID** | **Cluster no.** | ***No. of changes in expression level** | **time points tested** | **p-value** |
| --- | --- | --- | --- | --- |
| f10241_c0s2 | 11 | 3 | T1-T2 | 5.55E-03 |
|  |  |  | T2-T4 | 1.23E-01 |
|  |  |  | T2-T3 | 5.54E-01 |
| f12737_c0s1 | 33 | 3 | T1-T4 | 7.22E-04 |
|  |  |  | T1-T5 | 4.40E-03 |
|  |  |  | T1-T3 | 6.75E-03 |
| f14360_c0s1 | 33 | 1 | T1-T5 | 7.20E-04 |
| f14756_c0s4 | 28 | 6 | T3-T5 | 1.97E-02 |
|  |  |  | T3-T4 | 4.31E-02 |
|  |  |  | T2-T3 | 1.11E-01 |
|  |  |  | T1-T5 | 3.79E-01 |
|  |  |  | T1-T4 | 7.76E-01 |
|  |  |  | T1-T2 | 8.91E-01 |
| f14982_c0s1 | 4 | 4 | T2-T5 | 5.99E-08 |
|  |  |  | T4-T5 | 9.37E-08 |
|  |  |  | T3-T5 | 9.38E-08 |
|  |  |  | T1-T3 | 4.48E-04 |
| f16661_c0s1 | 20 | 1 | T3-T5 | 1.87E-02 |
| f19713_c0s1 | 42 | 2 | T1-T4 | 1.73E-02 |
|  |  |  | T2-T4 | 2.04E-01 |
| f22221_c0s1 | 20 | 4 | T3-T5 | 1.40E-02 |
|  |  |  | T4-T5 | 1.42E-02 |
|  |  |  | T2-T5 | 1.54E-02 |
|  |  |  | T1-T5 | 2.57E-02 |
| f24176_c0s3 | 20 | 1 | T3-T5 | 5.53E-01 |
| f2478_c0s1 | 4 | 5 | T3-T5 | 1.64E-02 |
|  |  |  | T2-T5 | 1.68E-02 |
|  |  |  | T1-T3 | 2.61E-02 |
|  |  |  | T1-T2 | 2.79E-02 |
|  |  |  | T4-T5 | 3.48E-02 |
| f26638_c0s7 | 11 | 2 | T4-T5 | 2.87E-02 |
|  |  |  | T3-T5 | 1.75E-01 |
| f3174_c0s2 | 33 | 3 | T1-T4 | 1.73E-07 |
|  |  |  | T1-T2 | 3.82E-05 |
|  |  |  | T1-T5 | 3.04E-03 |
| f5842_c0s1 | 7 | 4 | T1-T4 | 3.59E-07 |
|  |  |  | T4-T5 | 4.23E-07 |
|  |  |  | T1-T3 | 4.12E-04 |
|  |  |  | T3-T5 | 5.36E-04 |
| f614_c0s1 | 33 | 2 | T2-T5 | 2.22E-03 |
|  |  |  | T1-T5 | 9.05E-03 |
| f6864_c0s5 | 4 | 1 | T1-T3 | 2.65E-02 |
| f7423_c0s2 | 33 | 1 | T1-T4 | 2.46E-02 |
| f8322_c0s2 | 7 | 5 | T1-T4 | 2.94E-05 |
|  |  |  | T4-T5 | 2.97E-05 |
|  |  |  | T2-T4 | 3.19E-05 |
|  |  |  | T1-T3 | 1.37E-03 |
|  |  |  | T3-T5 | 1.48E-03 |
| f8633_c0s4 | 28 | 6 | T1-T4 | 9.03E-03 |
|  |  |  | T1-T5 | 9.98E-03 |
|  |  |  | T1-T2 | 2.74E-01 |
|  |  |  | T2-T3 | 2.90E-01 |
|  |  |  | T3-T4 | 3.58E-01 |
|  |  |  | T3-T5 | 4.48E-01 |
| f9861_c0s3 | 4 | 2 | T3-T5 | 1.16E-02 |
|  |  |  | T2-T3 | 4.47E-01 |
| v10022_cos1 | 3 | 1 | T4-T5 | 1.52E-01 |
| v11872_cos9 | 49 | 1 | T1-T5 | 2.92E-02 |
| v11959_c0s2 | 49 | 3 | T1-T4 | 4.04E-01 |
|  |  |  | T1-T3 | 4.20E-01 |
|  |  |  | T1-T5 | 6.59E-01 |
| v12185_c0s6 | 49 | 2 | T1-T4 | 1.33E-04 |
|  |  |  | T1-T3 | 4.55E-03 |
| v14961_c0s2 | 3 | 4 | T2-T4 | 4.16E-02 |
|  |  |  | T3-T4 | 3.17E-01 |
|  |  |  | T1-T4 | 6.21E-01 |
|  |  |  | T4-T5 | 8.50E-01 |
| v17835_c0s1 | 8 | 1 | T1-T2 | 1.34E-03 |
| v21389_c0s5 | 4 | 1 | T1-T2 | 6.59E-04 |
| v22201_c0s1 | 33 | 2 | T3-T5 | 1.90E-07 |
|  |  |  | T2-T5 | 4.71E-07 |
| v27339_c0s2 | 8 | 1 | T1-T2 | 1.38E-05 |
| v28401_c0s1 | 33 | 2 | T3-T5 | 1.07E-05 |
|  |  |  | T2-T5 | 1.36E-01 |
| v32335_c0s1 | 38 | 2 | T2-T4 | 1.58E-02 |
|  |  |  | T4-T5 | 2.82E-01 |
| v3401_c0s1 | 34 | 5 | T1-T4 | 7.42E-05 |
|  |  |  | T2-T4 | 4.57E-04 |
|  |  |  | T1-T5 | 5.33E-02 |
|  |  |  | T2-T5 | 9.71E-02 |
|  |  |  | T1-T3 | 1.00E+00 |
| v4137_c0s2 | 38 | 3 | T1-T3 | 6.97E-02 |
|  |  |  | T1-T2 | 1.13E-01 |
|  |  |  | T1-T5 | 3.26E-01 |
| v4845_c0s1 | 34 | 3 | T1-T4 | 1.17E-02 |
|  |  |  | T1-T5 | 6.61E-02 |
|  |  |  | T1-T3 | 4.83E-01 |
| v5438_c0s1 | 4 | 4 | T1-T2 | 2.00E-04 |
|  |  |  | T2-T3 | 1.09E-01 |
|  |  |  | T2-T5 | 1.23E-01 |
|  |  |  | T2-T4 | 1.69E-01 |
| v7121_c0s2 | 49 | 2 | T1-T3 | 1.03E-02 |
|  |  |  | T1-T4 | 7.31E-02 |
| v9357_c0s1 | 34 | 2 | T1-T3 | 4.22E-03 |
|  |  |  | T1-T5 | 5.34E-01 |
| v9648_c0s3 | 19 | 4 | T2-T5 | 5.72E-02 |
|  |  |  | T1-T5 | 2.24E-01 |
|  |  |  | T4-T5 | 2.28E-01 |
|  |  |  | T3-T5 | 3.37E-01 |
| v9750_c0s1 | 12 | 1 | T2-T3 | 1.74E-02 |

| total no. of tested sequences | 38 |  |
| --- | --- | --- |
| no. of sequences with no change confirmed as significant | 5 | 13.15% |
| no. of sequences with all changes confirmed as significant | 19 | 50% |
| no. of sequences with at least one change confirmed as significant | 14 | 86.85% |
