# Supplementary material for: Vernalization Mediated Changes in the Lolium perenne Transcriptome
Source: PLoS One. 2014 Sep 16;9(9):e107365. doi: 10.1371/journal.pone.0107365 (PMC4167334; doi:10.1371/journal.pone.0107365)
Supplement: Supplementary Material S5 — Individual expression profiles of the transcripts corresponding to the VRN1, GI, LHY, PRR37, CO9-LIKE, and galactinol-synthase genes observed in the leaves of Falster and Veyo genotypes. (DOCX) [file pone.0107365.s009.docx]

**Supplementary material 5**

Individual expression profiles for five key regulators of flowering time observed in the leaves of Falster and Veyo plants, two genotypes with contrasting vernalization requirement. The presented transcripts correspond to the VERNALIZATION 1 (VRN1), PSEUDRESPONSE REGULATOR 37 (PRR37), GIGANTEA (GI), LATE ELONGATED HYPOCOTYLE (LHY), CONSTANS 9-LIKE (CO9-LIKE) genes. The profiles observed for galactinol-synthase transcripts are also included in this material. The ‘x’ axis corresponds to the monitored time points. The ‘y’ axis corresponds to expression profiles, based on the variance stabilized data obtained from the DESeq software. Transcripts originating from Falster are presented in blue, while Veyo transcripts are presented in red and related shades. Solid lines represent transcripts identified as differentially expressed, while dotted lines represent transcripts which were not identified as differentially expressed in leaf samples using DESeq employing the function designed for no biological replicates.

In case of GI and LHY, a single transcript was present in the Falster transcriptome. However, Veyo transcriptome contained several transcripts corresponding to a fragmented transcript. These sequences show very similar expression patterns and reconstruct in length the Falster transcript.

The figure below represents the structure of the transcripts corresponding to the PRR37 gene. A genome scaffold from a preliminary draft assembly of the perennial ryegrass genome is illustrated.

v15408_c0s1

v15408_c0s2

f32885_c0s1

genomic scaffold

4962

5107

5200

5611

5693

6359

6473

6979

6511

81

bp

STOP codon

2,336 bp

2,417 bp

1,864 bp
